# Oral Microbiota Alteration and Roles in Epstein-Barr Virus Reactivation in Nasopharyngeal Carcinoma

**DOI:** 10.1128/spectrum.03448-22

**Published:** 2023-01-16

**Authors:** Ying Liao, Jiang-Bo Zhang, Li-Xia Lu, Yi-Jing Jia, Mei-Qi Zheng, Justine W. Debelius, Yong-Qiao He, Tong-Min Wang, Chang-Mi Deng, Xia-Ting Tong, Wen-Qiong Xue, Lian-Jing Cao, Zi-Yi Wu, Da-Wei Yang, Xiao-Hui Zheng, Xi-Zhao Li, Yan-Xia Wu, Lin Feng, Weimin Ye, Jianbing Mu, Wei-Hua Jia

**Affiliations:** a Sun Yat-Sen University Cancer Center, State Key Laboratory of Oncology in South China, Collaborative Innovation Center for Cancer Medicine, Guangdong Key Laboratory of Nasopharyngeal Carcinoma Diagnosis and Therapy, Guangzhou, China; b Department of Radiation Oncology, Sun Yat-Sen University Cancer Center, Guangzhou, China; c School of Public Health, Sun Yat-Sen University, Guangzhou, China; d Department of Medical Epidemiology and Biostatistics, Karolinska Institutet, Stockholm, Sweden; e Laboratory of Malaria and Vector Research, National Institute of Allergy and Infectious Diseases, National Institutes of Health, Rockville, Maryland, USA; Hubei University of Medicine

**Keywords:** oral microbiota, Epstein-Barr virus, nasopharyngeal carcinoma, microbiota-virus interaction, H_2_O_2_, *Streptococcus sanguinis*

## Abstract

Microbiota has recently emerged as a critical factor associated with multiple malignancies. Nasopharyngeal carcinoma (NPC) is highly associated with Epstein-Barr virus (EBV); the oncovirus resides and is transmitted in the oral cavity. However, the alternation of oral microbiota in NPC patients and its potential link to EBV reactivation and host cell response under the simultaneous existence of EBV and specific bacteria is largely unknown. Here, oral microbiota profiles of 303 NPC patients and controls with detailed clinical information, including serum EBV anti-virus capsid antigen (VCA) IgA level, were conducted. A distinct microbial community with lower diversity and imbalanced composition in NPC patients was observed. Notably, among enriched bacteria in patients, Streptococcus sanguinis was associated with anti-VCA IgA, an indicator of NPC risk and EBV reactivation. By measuring the concentration of its metabolite, hydrogen peroxide (H_2_O_2_), in the saliva of clinical patients, we found the detection rate of H_2_O_2_ was 2-fold increased compared to healthy controls. Further coculture assay of EBV-positive Akata cells with bacteria *in vitro* showed that S. sanguinis induced EBV lytic activation by its metabolite, H_2_O_2_. Host and EBV whole genome-wide transcriptome sequencing and EBV methylation assays showed that H_2_O_2_ triggered the host cell signaling pathways, notably tumor necrosis factor alpha (TNF-α) via NF-κB, and induced the demethylation of the global EBV genome and the expression of EBV lytic-associated genes, which could result in an increase of virus particle release and potential favorable events toward tumorigenesis. In brief, our study identified a characterized oral microbial profile in NPC patients and established a robust link between specific oral microbial alteration and switch of latency to lytic EBV infection status in the oral cavity, which provides novel insights into EBV’s productive cycle and might help to further clarify the etiology of NPC.

**IMPORTANCE** EBV is classified as the group I human carcinogen and is associated with multiple cancers, including NPC. The interplays between the microbiota and oncovirus in cancer development remain largely unknown. In this study, we investigate the interactions between resident microbes and EBV coexistence in the oral cavity of NPC patients. We identify a distinct oral microbial feature for NPC patients. Among NPC-enriched bacteria, we illustrated that a specific species, S. sanguinis, associated with elevated anti-IgA VCA in patients, induced EBV lytic activation by its by-product, H_2_O_2_, and activated the TNF-α/NF-κB pathway of EBV-positive B cells *in vitro*, together with increased detection rate of H_2_O_2_ in patients’ oral cavities, which strengthened the evidence of bacteria-virus-host interaction in physiological circumstances. The effects of imbalanced microbiota on the EBV latent-to-lytic switch in the oral cavity might create the likelihood of EBV infection in epithelial cells at the nasopharynx and help malignant transition and cancer development.

## INTRODUCTION

Nasopharyngeal carcinoma (NPC) is an epithelial malignancy originating from the nasopharynx. The estimated number of NPC cases was 129,000 worldwide in 2018 (1), and the majority of cases were reported in southern China and Southeast Asia (2). NPC and several lymphoid malignancies are consistently associated with Epstein-Barr virus (EBV) infection (3), which has been classified as a group I human carcinogen by the International Agency for Research on Cancer (4). Infection with this virus causes a considerable cancer burden, which accounts for approximately 300,000 cases of cancer each year (5, 6). As it is the most common virus infecting humans, over 90% of people worldwide are infected with EBV in childhood and sustain a persistent asymptomatic lifelong infection with periodic EBV shedding in the oral cavity. These viruses can be detected in the saliva of nearly 80% of individuals (7) and spread among individuals via saliva, which is the most common mode of EBV transmission.

Mounting evidence suggests that EBV infection, together with host genetic susceptibility, could drive NPC pathogenesis through multiple pathways, including epigenetic modification, immune evasion, and induction of genomic instability. As the major environmental factor in the oral-nasopharyngeal cavity, the oral microbiota has been associated with multiple cancers in a series of studies (8–12). The bacteria-derived metabolites have been proposed for their roles in cancer onset and progression (13, 14). Indeed, several opportunistic pathogens commonly observed in the oral community, such as Peptostreptococcus stomatis and Fusobacterium nucleatum, have recently been linked with various tumors from the head and neck region to the gastrointestinal tract (15, 16).

However, the characterization of oral microbiota in NPC is inadequately explored, and the possible influence of oral microbes on EBV infection status, as well as host cell responses, remains elusive. Although the link of EBV oncovirus role is unclarified between the oral and nasopharyngeal cavity, EBV abnormal behaviors in the oral cavity have frequently been reported in the increase of NPC risk (7) and unfavorable clinical outcome (17). To further clarify oral bacteria-EBV-host interplay roles in NPC patients, here, we performed 16S rRNA (rRNA) sequencing of saliva samples from 303 participants to characterize oral microbial changes in NPC patients. Using an *in vitro* coculture model, we illustrated the activation effect of S. sanguinis on EBV through its metabolite, H_2_O_2_. We showed that S. sanguinis coculture and H_2_O_2_ stimulation correlate with the activation of signaling pathways, including tumor necrosis factor alpha (TNF-α) via NF-κB in the host cells, the upregulation of EBV-carrying genes, and the demethylation of the EBV genome.

## RESULTS

### NPC patients have distinct oral microbial communities.

**FIG 1:**
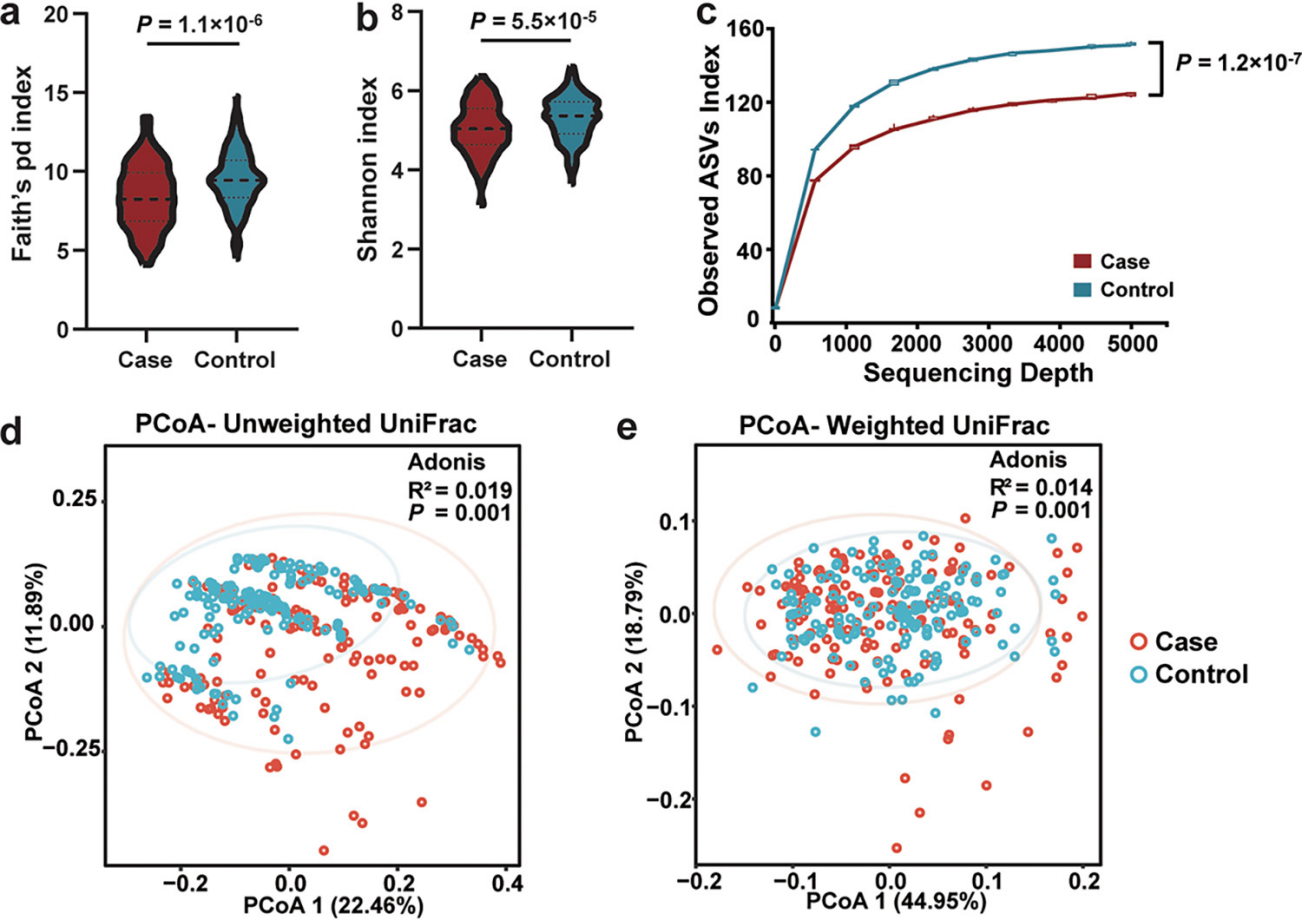
Distinct oral microbial diversity and composition between NPC patients and controls. (a to c) NPC patients have a lower-diversity microbiota. Alpha diversity calculated with Faith’s pd index (a) and the Shannon index (b) are shown in the box plots, and the observed ASV index (c) is shown in a rarefaction plot. *P* values were determined by logistic regression adjusting for age, sex, education, smoking status, alcohol consumption, oral health status (including three variables of teeth loss after age 20, filling teeth, and tooth brushing frequency), sequencing depth, and experimental batch. (d and e) Principal-coordinate analysis (PCoA) plots showing the distinct microbial composition between NPC patients and controls based on unweighted UniFrac distance (d) and weighted UniFrac distance (e). The *P* value and *R*^2^ were determined by Adonis analysis adjusting for age, sex, education, smoking status, alcohol consumption, oral health status, sequencing depth, and experimental batch. Shown are 95% confidence ellipses.

To identify ecological changes associated with NPC, we assessed the oral microbiota diversity and composition differences between NPC patients and controls. Participants’ demographics and characteristics are shown in Table S1 in the supplemental material. Lifestyle, including cigarette smoking status and oral health status, was found to have significant effects on microbial diversity (Table S2). These variables, combined with other potential confounders (age, sex, education, and alcohol consumption), were adjusted in the following analyses. We found a significant decrease in the Faith phylogenetic diversity (Faith’s pd) index (Fig. 1a) and Shannon index (Fig. 1b) in NPC patients compared to healthy controls (logistic regression, *P = *1.1 × 10^−6^ and *P = *5.5 × 10^−5^, respectively). Similarly, the richness of the oral microbiota, represented by the observed amplicon sequence variants (observed_ASVs) index, was also significantly decreased in NPC patients (logistic regression, *P = *1.2 × 10^−7^) (Fig. 1c). This is consistent with a previous report that NPC patients had a lower diversity of oral microbiota (18). Next, we assessed the microbial community structure differences between NPC patients and controls. The results showed that oral microbial communities were significantly influenced by disease status in unweighted UniFrac distances (Adonis *R*^2^ = 0.019, *P = *0.001) (Fig. 1d) and weighted UniFrac distances (Adonis *R*^2^ = 0.014, *P = *0.001) (Fig. 1e). These results indicated that the oral communities of NPC patients were distinct from those of controls.

### Notable taxonomic alterations in oral microbiota between NPC patients and controls.

In the oral profiling analysis, bacterial taxa were identified as amplicon sequence variants (ASVs) by the DADA2 method. We performed DESeq analysis of the 219 core taxa that were present in more than 20% of individuals to identify NPC-associated features. The results showed that the relative abundances of 10 taxa were significantly different in NPC patients from controls, with a false-discovery rate (FDR) correlation of *P*-adj values of <0.05 (Table 1). Six of the 10 significantly different taxa were enriched in NPC patients, namely, Lautropia mirabilis ASV.c845 (*P = *6.82 × 10^−7^), Oribacterium asaccharolyticum ASV.37ab (*P = *6.44 × 10^−5^), *Actinomyces* sp. strain ASV.5a26 (*P = *4.44 × 10^−4^), Capnocytophaga sputigena_ASV.ce54, (*P = *9.27 × 10^−4^), Streptococcus sp. strain ASV.1b51 (*P = *1.56 × 10^−3^), and Peptostreptococcus stomatis ASV.1f05 (*P = *1.50 × 10^−3^) (Table 1). Three taxa were found to be depleted in NPC patients, namely, *Bacteroidetes* HMT 511 ASV.021c (*P = *8.03 × 10^−5^), TM7 HMT 356 ASV.3216 (*P = *5.31 × 10^−5^), *Leptotrichia* sp. strain ASV.e811 (*P = *8.10 × 10^−5^), and TM7 HMT 351 ASV.fcb6 (*P = *1.63 × 10^−3^) (Table 1). The relative abundances, presence rates, and DESeq results of 45 core taxa with *P* values of <0.05 are shown in Table S3.

**TABLE 1:** Relative abundance and presence rate difference of oral microbiome between NPC patients and controls^*a*^

Taxonomy	Relative abundance (%)		Presence rate (%)		Log_2_Fc (95% CI)	*P* value
	Case	Control	Case	Control		
	(*n* = 150)	(*n* = 153)	(*n* = 150)	(*n* = 153)		
NPC-enriched taxa						
*Lautropia mirabilis* ASV.c845	1.47	0.80	95.33	96.08	1.07 (0.86, 1.29)	6.82 × 10^−7^
*Oribacterium asaccharolyticum* ASV.37ab	0.12	0.05	45.33	36.60	1.73 (1.30, 2.17)	6.44 × 10^−5^
*Actinomyces* ASV.5a26	0.89	0.42	30.00	21.57	2.06 (1.47, 2.64)	4.44 × 10^−4^
*Capnocytophaga sputigena* ASV.ce54	0.40	0.30	84.00	86.27	0.74 (0.52, 0.97)	9.27 × 10^−4^
Streptococcus ASV.1b51	0.52	0.33	74.67	64.71	0.89 (0.61, 1.17)	1.56 × 10^−3^
Peptostreptococcus stomatis ASV.1f05	0.27	0.18	89.33	96.08	0.67 (0.46, 0.88)	1.50 × 10^−3^
NPC-depleted taxa						
*Bacteroidetes* HMT 511 ASV.021c	0.09	0.22	24.67	45.75	−1.62 (−2.03, −1.21)	8.03 × 10^−5^
TM7 HMT 356 ASV.3216	0.04	0.08	36.00	61.44	−1.38 (−1.72, −1.04)	5.31 × 10^−5^
*Leptotrichia* ASV.e811	0.07	0.16	30.67	63.40	−1.45 (−1.82, −1.08)	8.10 × 10^−5^
TM7 HMT 351 ASV.fcb6	0.71	1.22	51.33	79.08	−0.88 (−1.15, −0.60)	1.63 × 10^−3^

a We analyzed 219 core taxa with a detection rate >20%, and they were adjusted for age, sex, education, smoking status, alcohol consumption, and oral health status (including three variables of teeth loss after age 20, filling teeth, and tooth brushing frequency); 10 ASVs with *P-*adj values of <0.05 in DESeq analysis are shown. The first 4 letters of the ASV ID are shown in the taxonomy. CI, confidence interval.

To identify the species information of Streptococcus_ASV.1b51, we performed quantitative PCR (qPCR) with species-specific primers to detect the top-hit Streptococcus spp. in the NCBI BLAST database. We noticed that Streptococcus sanguinis was the only species with 100% similarity from the NCBI database (Table S4). Consistently, qPCR results revealed that the relative abundance of Streptococcus ASV.1b51 detected by 16S rRNA sequencing was highly correlated with the relative abundance measured by species-specific primers of S. sanguinis (Spearman *R* = 0.80, *P < *0.0001) (Fig. S2) but not with the abundances of the other four candidate species (data not shown). We inferred that a large proportion of Streptococcus ASV.1b51, if not the whole amount, was S. sanguinis in the samples from this study.

### The association between NPC-associated microbial features and serum EBV VCA-IgA antibody levels.

**FIG 2:**
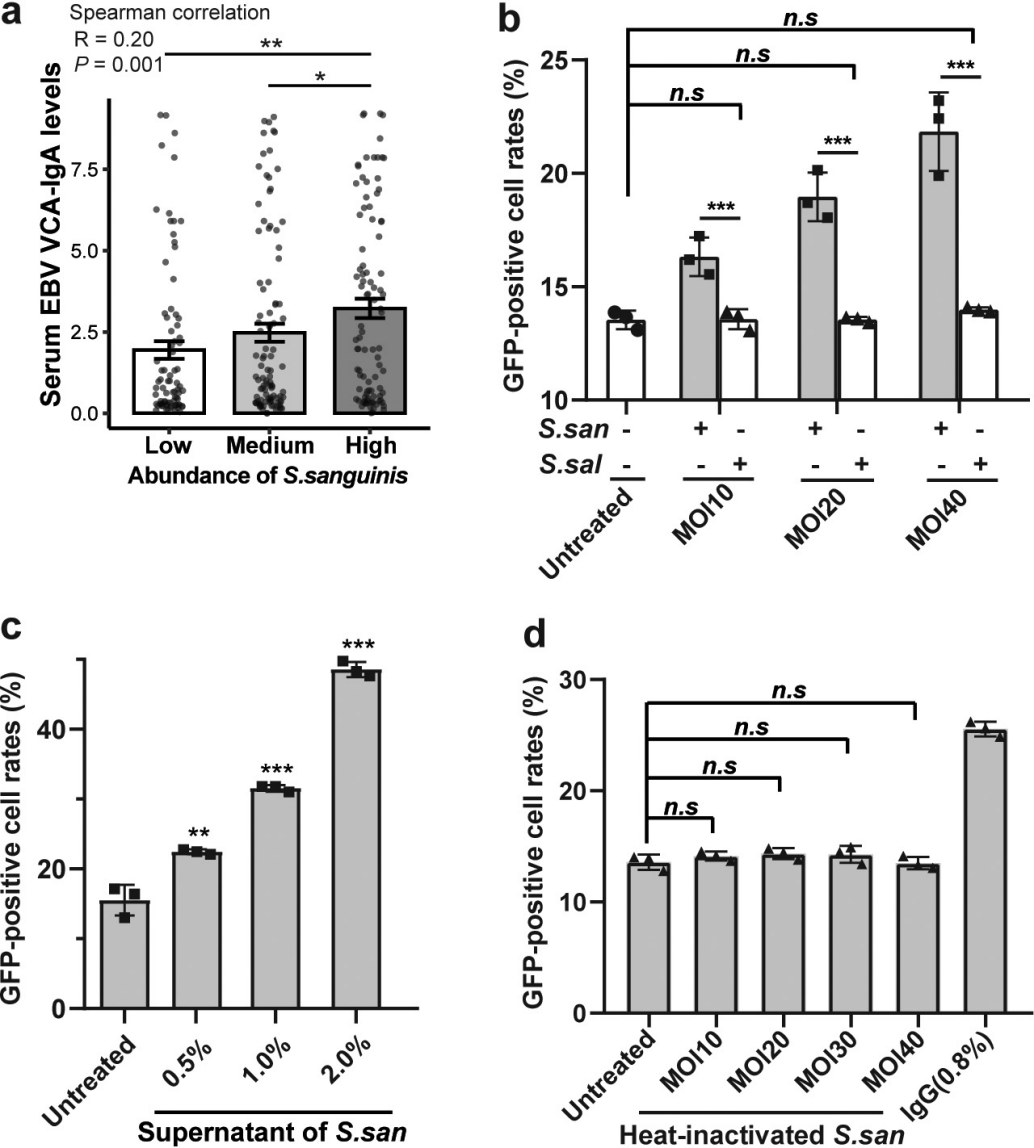
Streptococcus sanguinis induces EBV activation. (a) Association between the abundance of S. sanguinis and serum EBV VCA-IgA levels. *P* values of comparison between the two groups were determined by Wilcox tests (two-tailed). (b) Flow cytometry detection of GFP-positive cell rates of Akata cells cocultured with Streptococcus sanguinis (*S. san*) and Streptococcus salivarius (*S. sal*); MOI = 10, MOI = 20, and MOI = 40 indicated that the multiplicity of infection (MOI) values of bacterial infection of Akata cells were 10, 20, and 40, respectively. (c) Flow cytometry detection of GFP-positive cell rates of Akata cells treated with supernatant of Streptococcus sanguinis. (d) Flow cytometry detection of GFP-positive cell rates of Akata cells treated with heat-inactivated Streptococcus sanguinis; IgG (0.8%) was used as the positive treatment. Each column represents 3 independent biological replicates. *P* values were determined by unpaired *t* tests (two-tailed). *, *P < *0.05; **, *P < *0.01; ***, *P < *0.001; ns, not significant.

EBV resides and is transmitted in the oral cavity; its infection is closely associated with NPC. Elevated biomarkers indicative of EBV reactivation, such as serum EBV virus capsid antigen (VCA)-IgA, have been reported as high-risk factors for NPC in several epidemiological studies (19, 20). As the main microenvironment of the oral-nasopharyngeal sites, it has attracted our attention over whether the oral microbiota plays a role in the EBV infection status of the host. By evaluating the association between NPC-enriched oral microbes and serum EBV-VCA IgA antibody levels, we found that moderate but significant association between the abundance of Streptococcus sanguinis ASV1b51 and serum EBV VCA-IgA antibody levels (Spearman correlation, *R* = 0.20, *P = *0.001) (Fig. 2a). Associations with serum EBV VCA-IgA antibody levels were also observed for the abundance of Oribacterium asaccharolyticum ASV.37ab and Capnocytophaga sputigena ASV.ce54 (Spearman correlation, *R* = 0.14, *P = *0.022; *R* = 0.13, *P = *0.037, respectively) (Fig. S3a and b). These results indicated that NPC-enriched microbes might be involved in EBV infection in hosts.

### Streptococcus sanguinis induces EBV lytic activation.

To validate the association between S. sanguinis and EBV reactivation, we applied an *in vitro* coculture model with EBV-positive cells and S. sanguinis. We chose Akata cells, a B lymphocyte cell line carrying a green fluorescent protein (GFP)-tagged recombinant EBV strain (21), because B lymphocytes are the main reservoir of EBV in the oral microenvironment. The activation status of EBV was measured by the GFP-positive rates of cocultured Akata cells using flow cytometry and EBV copy numbers of the coculture systems. Streptococcus salivarius was applied as a control taxon in *in vitro* experiments. We found that the coculture of S. sanguinis induced significant GFP-positive rates of Akata cells in a dose-dependent manner, while coculture with S. salivarius did not induce EBV reactivation in host cells, even in the high-dosage MOI40 group (Fig. 2b). We next investigated if exposure to the supernatant or bacterial body of S. sanguinis can induce cellular EBV reactivation. We found that the culture supernatant of S. sanguinis could significantly induce cellular EBV reactivation (Fig. 2c). However, the heat-inactivated S. sanguinis did not show the EBV reactivation effect on cells (Fig. 2d). These results indicated that S. sanguinis could induce cellular EBV reactivation, which might be attributed to its by-product released into the culture supernatant.

### Streptococcus sanguinis induces EBV lytic activation by its metabolite, hydrogen peroxide.

**FIG 3:**
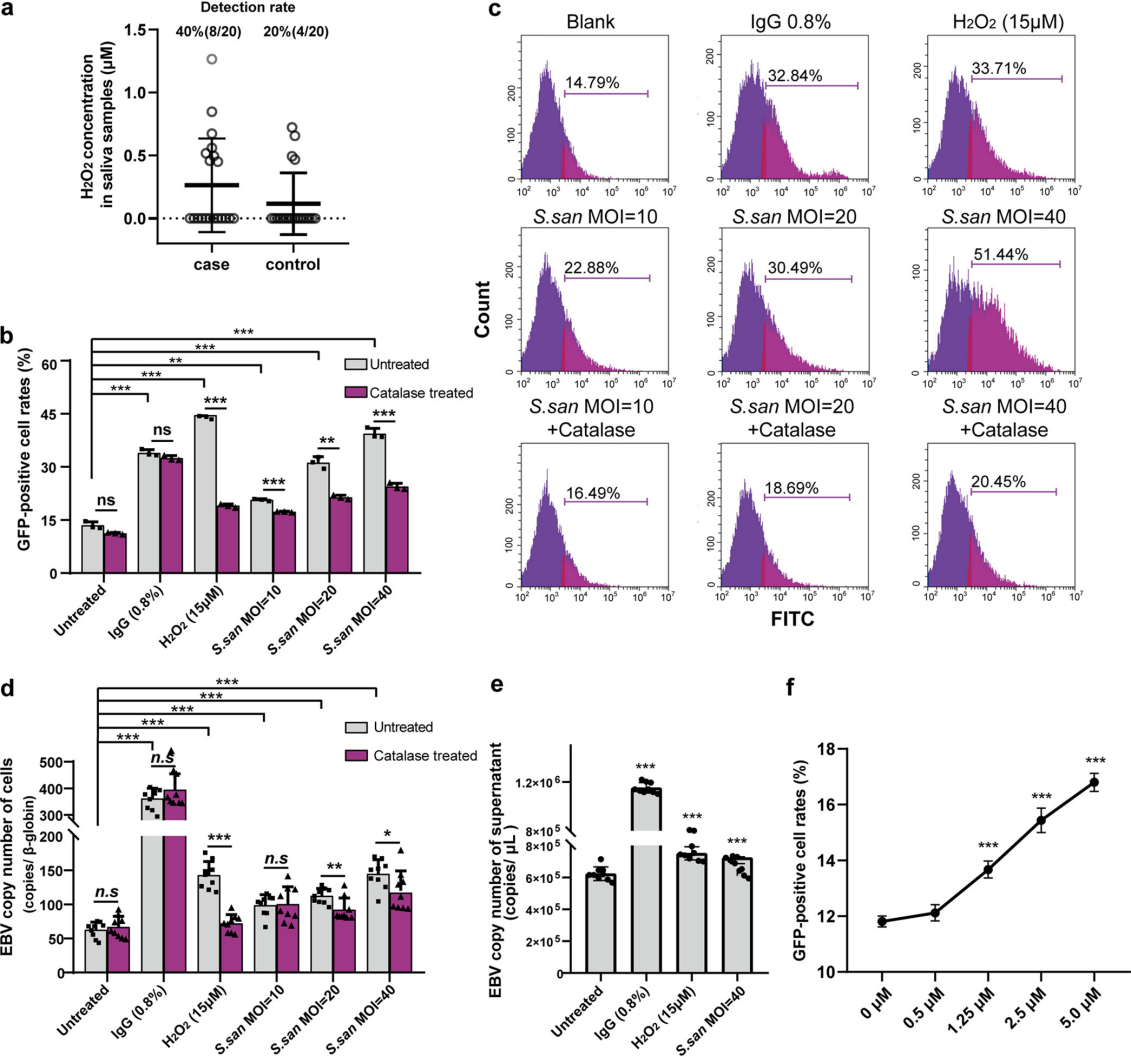
The EBV-inducing effect of Streptococcus sanguinis depends on its secreted H_2_O_2._ (a) Levels of H_2_O_2_ in the saliva of 20 NPC patients and 20 healthy controls. (b) Flow cytometry detection of GFP-positive cell rates of Akata cells cocultured with Streptococcus sanguinis (*S. san*), treated with H_2_O_2_ and IgG. (c) Representative results of flow cytometry detection. (d and e) EBV DNA copy number detection of the cells (d) and the supernatant (e) of the coculture system. IgG (0.8%) was used as the positive treatment. Catalase was added to the culture system at a final concentration of 200 units/mL to eliminate H_2_O_2_. Each column represents 3 independent biological replicates. (f) Flow cytometry detection of GFP-positive cell rates of H_2_O_2_-treated cells. Akata cells were treated with 0 μM, 0.5 μM, 1.25 μM, 2.5 μM, and 5.0 μM H_2_O_2_ every 2 h, and cells were harvested at 24 h. Data are expressed as the mean ± SD. *P* values were determined by unpaired *t* tests (two-tailed). *, *P < *0.05; **, *P < *0.01; ***, *P < *0.001; ns, not significant.

S. sanguinis is a commensal bacterium in the oral cavity and can produce hydrogen peroxide (H_2_O_2_) (22). Elevated reactive oxygen has been described to play a role in viral carcinogenesis in EBV-associated cancers (23). We next investigated whether enriched S. sanguinis in NPC patients’ oral cavities affect oral EBV biology through its metabolite, H_2_O_2_. We first measured the concentration of H_2_O_2_ in saliva samples from NPC cases and controls. The detection rate of H_2_O_2_ in the saliva of NPC patients was twice as high as that of healthy controls, 40% in NPC and 20% in controls, respectively, (Fig. 3a). We then measured the H_2_O_2_ concentration in S. sanguinis culture supernatants and confirmed the increasing H_2_O_2_ concentration with S. sanguinis density (Fig. S4). Interestingly, coculture of S. sanguinis with Akata cells significantly induced the lytic activation of EBV, while catalase treatment abolished the activation effect of H_2_O_2_ and S. sanguinis (Fig. 3b and c). To confirm the EBV activation results, we determined the EBV DNA copy number of the cells and supernatant of the coculture systems. The coculture of S. sanguinis with Akata cells significantly induced the increase of EBV DNA copy number, which was abolished by catalase treatment (Fig. 3d). EBV copy number of the supernatant also significantly elevated with the coculture of S. sanguinis (Fig. 3e). Collectively, these results demonstrated that S. sanguinis induces the lytic activation of EBV mainly through its H_2_O_2_ production.

To better simulate the physiological conditions, that is, the sustained effect of low-dose H_2_O_2_, we treated cells with a series of concentrations of H_2_O_2_ every 2 h (the degradation time of H_2_O_2_ when coculturing, shown in Fig. S5) and harvested cells at 24 h to detect the EBV-positive rate with flow cytometry. We found that H_2_O_2_ significantly upregulated EBV lytic activation at a low concentration of 1.25 μM (Fig. 3f), which is comparable with the H_2_O_2_ concentration in NPC patients’ saliva.

### Streptococcus sanguinis alters the transcription of the genes in host cells and EBV.

**FIG 4:**
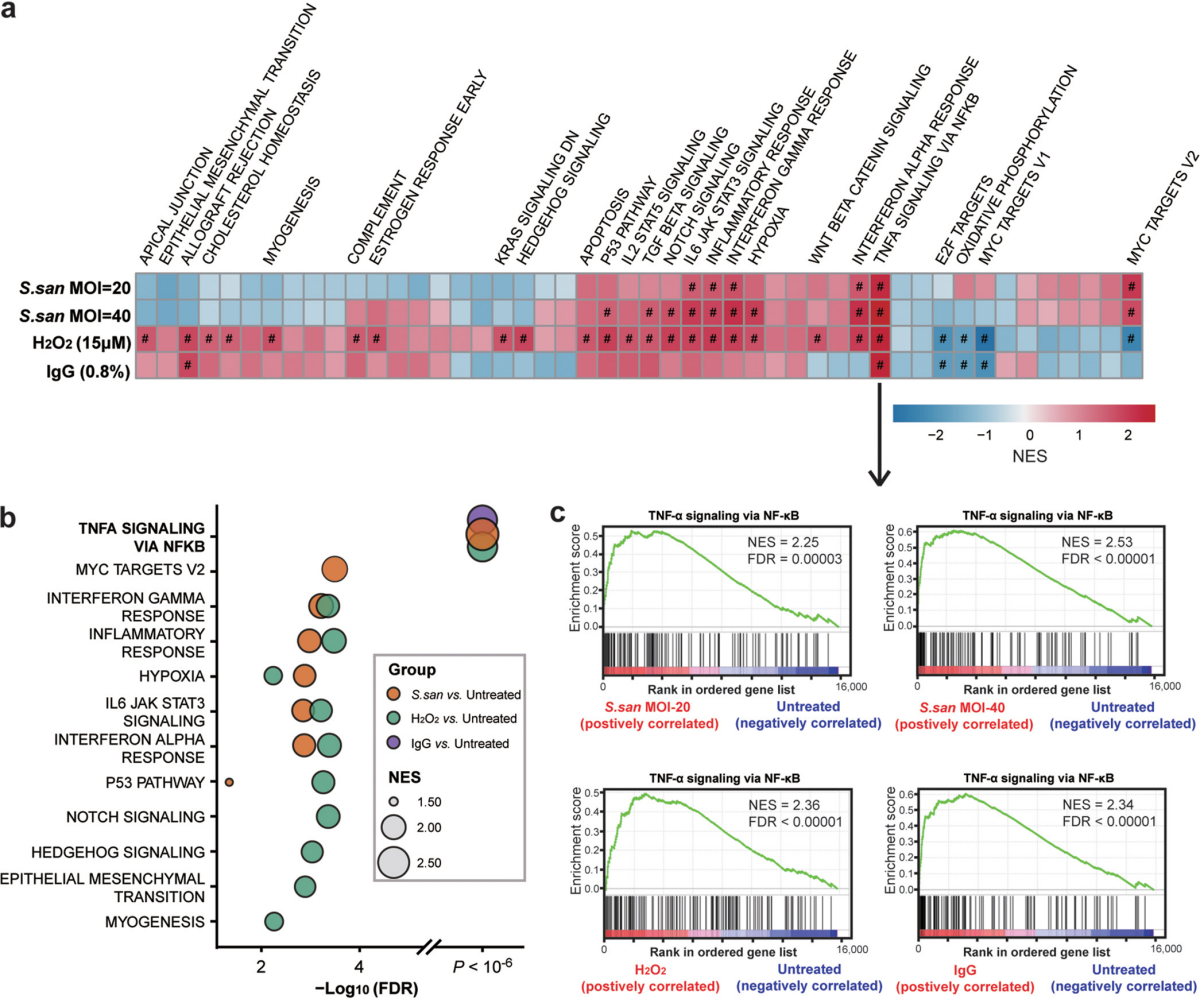
Whole-transcriptome sequencing reveals that Streptococcus sanguinis coculture with EBV-infected Akata cells induces specific cellular signaling pathways. (a) Heatmap shows enrichment scores of Streptococcus sanguinis (*S. san*) MOI of 20, S. sanguinis MOI of 40, and H_2_O_2_- and IgG-treated models using the 48 hallmark pathways from Molecular Signatures DataBases (MSigDB) from gene set enrichment analysis (GSEA). Pathways with a false-discovery rate (FDR) *q* value of <0.05 are annotated with “#” and labeled with pathway names. Detailed normalized enrichment scores (NESs), FDR *q* values, and core enrichment genes of significant pathways are shown in Table S6 in the supplemental material. (b) Significantly enriched signaling pathways in the S. sanguinis*-*, H_2_O_2_-, and IgG-treated groups versus the untreated group. Data of S. sanguinis MOI of 20 and S. sanguinis MOI of 40 were calculated by categorization into the S. sanguinis group. Signaling pathways with an FDR *q* value of <0.05 are shown (for the H_2_O_2_-treated group versus the untreated group, the top 10 significantly enriched pathways are shown). (c) GSEA enrichment plots of TNF-α via the NF-κB hallmark pathway of S. sanguinis MOI of 20, S. sanguinis MOI of 40, and H_2_O_2_- and IgG-treated groups with NES and FDR.

To better understand the effects of S. sanguinis on host cells and EBV, we performed transcriptome sequencing (RNA-seq) analysis for Akata cells cocultured with S. sanguinis and treated with H_2_O_2_. IgG treatment data were obtained from the study of O’Grady et al. (24). The results of the gene set enrichment analysis (GSEA) showed a significant overlap of enriched signaling pathways in response to different stimuli (Fig. 4a and b and Table S5). The top enriched signaling pathway, TNF-α via the NF-κB pathway, showed the largest normalized enrichment score (NES) in untreated versus S. sanguinis multiplicity of infection (MOI) of 20, S. sanguinis MOI of 40, H_2_O_2_, and IgG groups (NES = 2.25, 2.53, 2.36, and 2.34, respectively) (Fig. 4c). Genes from this signaling pathway, such as *NFκB1*, *NFκBIA*, *NFκB2*, *RELB*, *TNFAIP3*, and *TNFSF9*, were core enriched in the coculture group (Table S5). Other enriched pathways shared between the S. sanguinis coculture group and the H_2_O_2_ treatment group included interferon gamma response, interferon alpha response, inflammatory response, and interleukin 6 (IL-6)/JAK/STAT3 signaling (Fig. 4a and b). These results indicated that S. sanguinis coculture and H_2_O_2_ treatment induced a similar molecular response in host cells, suggesting that the EBV-inducing effects of S. sanguinis were mainly from its metabolite, H_2_O_2_, which was consistent with results from *in vitro* assay.

**FIG 5:**
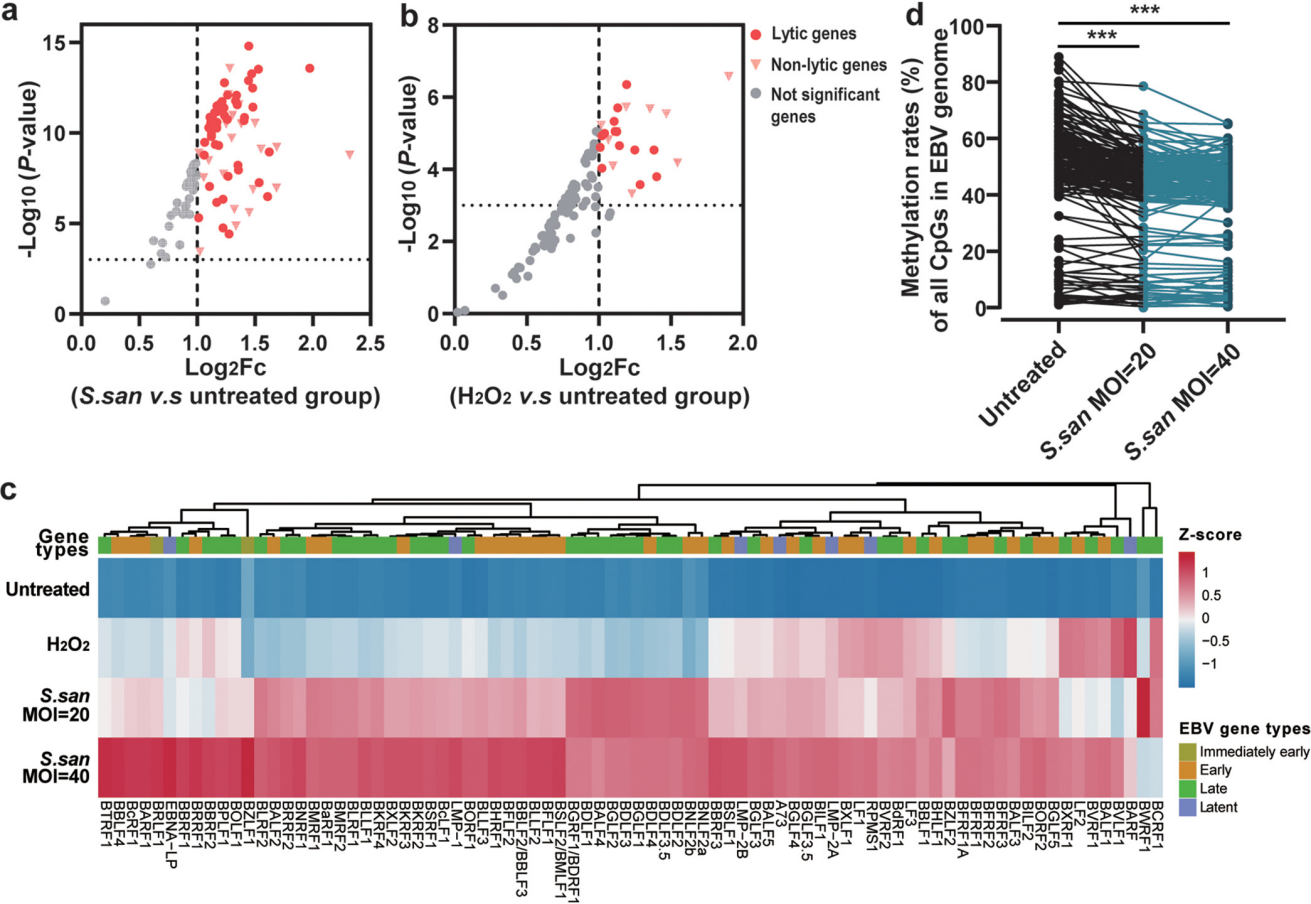
Streptococcus sanguinis coculture induces global upregulation of gene expression and whole-genome demethylation of EBV. (a and b) Volcano plots highlighting the upregulation of EBV gene expression in the S. sanguinis coculture group (a) and H_2_O_2_-treated group (b). EBV expression data were obtained by RNA-seq. Log_2_ fold change (Log_2_Fc) and *P* values were determined by edgeR. Significant gene thresholds are absolute values of Log_2_Fc of ≥1 and FDR *q* value of <0.05. (c) Heatmap of EBV gene expression in S. sanguinis-cocultured and H_2_O_2_-treated Akata cells. All EBV genes annotated to immediately early (*n* = 2), early (*n* = 31), late (*n* = 40), and latent (*n* = 7) types are shown. Z-scores of TMM-normalized gene expression counts were scaled by row. Hierarchical clustering was performed on both the rows and the columns with clustering metrics (“Euclidean”) and methods (“complete”). (d) Methylation rates of all annotated CpG sites (*n* = 183) in EBV genomes were generally decreased in S. sanguinis-cocultured Akata cells compared with untreated Akata cells (see Table S8 in the supplemental material). *P* values were determined by paired *t* tests (two-tailed); degrees of freedom (df), 182. ***, *P < *0.001. *S. san*, Streptococcus sanguinis.

With the coculture of S. sanguinis, we also observed a global upregulation of the EBV-carrying genes, especially lytic genes (Fig. 5a and c and Table S6), indicating the lytic activation of EBV. The results from independent validation of qPCR detection also showed significantly higher expression of EBV genes in the H_2_O_2_-treated and S. sanguinis coculture groups (Fig. S6). Since the expression of EBV genes is strictly regulated through the methylation of EBV DNA, we further explored the methylation alterations of the EBV genome caused by S. sanguinis coculture. In accordance with the changes in the virus gene expression pattern, we observed demethylation of the whole genome of EBV under the coculture of Akata cells with S. sanguinis (Fig. 5d), especially for the genome regions carrying early and late genes (Fig. S7 and Table S7).

## DISCUSSION

Here, we studied the interactions between resident microbes and EBV coexistence in the oral cavity, focusing on how specific species induce EBV reactivation standing, by providing population-based evidence, clinical association, and *in vitro* experiments with omics sequencing data. In particular, NPC patients harbored distinct oral microbiota with significantly lower diversity and imbalanced composition than healthy controls. We identified S. sanguinis, an enriched taxon in NPC patients and associated with serum EBV VCA-IgA level, can induce EBV lytic activation in EBV-positive cells, mainly through the production of H_2_O_2_ *in vitro*. Whole-transcriptome and EBV-targeted methylation sequencing further demonstrated that the coculture of S. sanguinis with EBV-infected Akata cells affects the host signaling pathways, mainly TNF-α and NF-κB, and upregulates EBV-carrying genes and demethylates the EBV genome. These findings expand our understanding of the interactions between oral microbiota and EBV life behavior, as well as host cell responses, providing a new perspective to clarify the etiology of NPC.

Recent studies have reported that oral microbiota might be the indicators for the diagnosis, prognosis, and clinical therapy side effects of NPC. Debelius et al. (25) reported lower community richness and altered community structures and found that subspecies niche specialization of Granulicatella adiacens in the oral microbiota was associated with NPC risk. Du et al. (26) reported the association of lower diversity of oral microbiota with higher mortality of NPC. Several studies have reported the association between oral microbiota and the progression of oral mucositis in NPC patients during radiotherapy (18, 27). All of these studies illustrated the potential roles microbiota played in the tumorigenesis and progression of NPC.

There is a current view that EBV lytic infection might contribute to the expanding EBV load and condition of the tumor microenvironment, thereby participating in EBV-derived tumor formation (28–30). EBV lytic reactivation contributes to primary infection and the establishment of latency in the host (28). Indeed, our previous epidemiological studies have shown the association between oral viral load and NPC risk: the saliva EBV DNA load was 10.2- to 67.6-fold higher in individuals from NPC areas of endemicity than those from non-areas of endemicity (7); the oral EBV DNA load was also observed to be independently associated with worse overall survival of NPC patients (hazard ratio [HR] = 1.45) (17). Additionally, evidence from our previous study and others have demonstrated that serum EBV VCA-IgA, an indicator of EBV activation, preceded long before the development of NPC (31, 32), together highlighting the etiological role of continuous EBV reactivation in NPC development. It is more likely that lytic reactivation leads to an elevated viral load in the host and expansion of EBV latently infected B lymphocyte populations, which, in turn, increases the likelihood of establishing latent infection in the nasopharyngeal epithelium and thereby increasing the possibility of developing NPC.

The oral cavity is the primary infection and final release site for EBV transmission in which a wide range of bacteria are nurtured and interacted with the microenvironment of the oronasopharynx. Thus, studies on the relationship between oral microbes and EBV are necessary but lacking. In this study, we observed that the H_2_O_2_-producing bacterium S. sanguinis was significantly enriched in the oral microbiota of NPC patients and associated with serum EBV VCA-IgA levels, coupled with the elevated H_2_O_2_ detection rate in patients’ saliva. To the best of our knowledge, it is the first time reporting the specific bacterium both associated with NPC and EBV reactivation. Previous studies have reported that some oral pathogens, such as Porphyromonas endodontalis and Aggregatibacter actinomycetemcomitans, could cause EBV reactivation in latently infected cells *in vitro* (33, 34), outlining a critical role of oral microbes in EBV infection, but they lacked sufficient population data to support their association with NPC. S. sanguinis is a Gram-positive coccus, formerly known as Streptococcus sanguis, commonly found in the oral cavity. Sakamoto et al. (35) found that oral Streptococcus spp., including S. sanguinis, were the most common isolates from cervical lymph nodes in patients with oral cancer, which indicated the potential role of this genus in the carcinogenesis of head and neck cancer (36).

Interestingly, S. sanguinis has a great capacity to produce H_2_O_2_ via the enzymatic reaction catalyzed by its pyruvate oxidase, SpxB. Recent studies have shown the role of oxidative stress and H_2_O_2_ in EBV reactivation (37). H_2_O_2_ and other reactive oxygen species can mediate NF-κB activation by proinflammatory cytokines, such as TNF-α (38). Constitutive activation of NF-κB is essential for the tumorigenesis of EBV-positive carcinomas, such as nasopharyngeal carcinoma (39) and EBV-infected lymphomas (40). Consistent with these studies, we found that coculture of S. sanguinis or direct H_2_O_2_ stimulation of EBV-infected Akata cells can significantly demethylate the EBV genome and alter the host cell and EBV gene expression. Further studies on how oral microbiota regulate EBV life behavior are still needed, which should provide broader evidence for the etiological exploration and prevention strategies for a wide range of EBV-associated malignancies.

In addition to S. sanguinis, four other taxa annotated with species-level information were found to be enriched in NPC patients. (i) Peptostreptococcus stomatis ASV.1f05 (P. stomatis), which is an anaerobic Gram-negative coccus and periodontal pathogen, has been found to be one of the top enriched taxa in both colorectal and gastric tumor tissues (15, 16). This microorganism was reported to promote colorectal carcinoma formation and progression by binding to integrin α2/β, activating the PI3K-Akt pathway and NF-κB, and modulating tumor immunity (41). This taxon is also overrepresented in the oral microbiota of oral squamous cell cancer patients (42). (ii) Capnocytophaga sputigena ASV.ce54 (C. sputigena), a Gram-negative motile bacillus, has been reported to be pathogenic in periodontal diseases and opportunistic infections (43). The relative abundance of oral *Capnocytophaga*, the genus of C. sputigena, has been associated with tumor recurrence in the oral microbiota of oral squamous cell cancer patients (44). The two other enriched taxa were (iii) Lautropia mirabilis ASV.c845 (L. mirabilis), which is a Gram-negative facultatively anaerobic motile coccus, and (iv) Oribacterium asaccharolyticum ASV.37ab (O. asaccharolyticum), which is a Gram-positive, strict anaerobic motile rod. There are limited data on the pathogenicity of these two taxa. The pathogenic roles of other NPC-enriched bacteria in NPC development remain to be further investigated.

Taxa belonging to TM7, *Bacteroidetes* HMT 511, and the genus *Leptotrichia* were observed depleted in NPC patients. TM7 (*Saccharibacteria*) are epibionts colonized on the surface of their host bacteria, which are commonly observed in the host oral cavity. One recent study reported its protective roles in reducing oral inflammation and consequential bone loss by mediating the downregulation of host bacterial pathogenicity (45). Bacteroidetes_[G-5], the genus of *Bacteroidetes* HMT 511_ASV.021c, was reported to be depleted in the mouth rinse samples of lung cancer patients, indicating its possible connections with lower cancer risk (46). The higher abundance of the genus *Leptotrichia* in oral was previously reported to be associated with decreased pancreatic cancer risk (9) and also linked with less bone loss around nonsubmerged implants during healing (47). These studies together indicated the potential protective roles these taxa might play in the carcinogenesis of NPC, which provides evidence for the development of the oral microbiome-based preventive approach to NPC.

In the current study, we focused on the association between oral microbiota and NPC, considering the huge differences in the microbiota between oral and nasopharyngeal sites (48); studies of the nasopharyngeal microbiota of NPC are still needed, which could help clarify the role of microbiota *in situ* in the development of NPC. Also, due to the short read lengths obtained from the 16S rRNA-based next-generation sequencing, the species annotation information is insufficient, further studies based on metagenomic or full-length 16S rRNA gene sequencing will allow us to get a more precise identification of the oral microbial community. Moreover, as associations have been found between microbiota and clinical responses to clinical therapy (49, 50), studies on the oral microbial profile alteration in response to the NPC treatment are still warranted. Outliers issues are also needed to take into consideration when performing microbiome analysis. Detection of microbiome outliers is still challenging because microbiome data often widely vary between individuals, and it is not easy to define the “normal” composition of the microbiome data. Some works have reported the outlier detection methods for microbiome data, which provide a good attempt for related research (51); further methodological studies with larger test data are still required to develop more precise and applicable tools.

In summary, our study focused on the alteration of oral microbiota in NPC patients, uncovered a robust link between oral microbial alteration profile and EBV latent-to-lytic infection in the oral cavity, and depicted the host cell response with the coexistence of microbes and EBV. Our study delineates a scenario where NPC-associated oral microbiotas directly induce EBV reactivation, which improves our understanding of the role oral microbiota play in EBV’s productive cycle and possibly in NPC development.

## MATERIALS AND METHODS

### Sample collection.

This study was approved by the Human Ethics Committee of Sun Yat-Sen University Cancer Center (SYSUCC) (approval numbers GZR2013-008 and GZR2019-217). An informed consent form was signed by every subject before the interview. We recruited 186 NPC patients and 153 healthy controls from February 2013 to July 2017 from Guangdong Province, China. NPC patients were recruited from SYSUCC, and all patients were histopathologically confirmed. Healthy controls were self-reported cancer-free individuals recruited from the physical examination center at the same time, which have been previously described in detail (52). A subset of 150 NPC patients who were newly diagnosed and 153 healthy controls who were matched to the patients by age and sex were used to identify the alteration of oral microbiota in NPC patients. Questionnaires, saliva, and blood specimens were collected during the interview. Demographics of the case-control study participants are presented in Table S1 in the supplemental material.

Saliva samples were collected from each participant for DNA extraction. Participants were instructed to avoid eating, smoking, and chewing gum for 30 min, or drinking water for 15 min, prior to saliva collection. Unstimulated saliva was collected by asking the subjects to spit saliva into a sterile tube. Collected saliva was immediately placed on ice and then aliquoted and stored at −80°C for 4 h. A 5-mL blood sample was collected from each participant. Whole blood was left undisturbed at room temperature for 1 to 2 h. The serum was collected by centrifuging the whole blood at 3,500 rpm for 15 min.

### DNA extraction and 16S rRNA V4 region sequencing.

All samples were randomized before DNA extraction to prevent batch effects. Total DNA from 400-μL saliva samples was extracted using the DNeasy PowerSoil kit (Qiagen, Duesseldorf, Hilden, Germany) following the manufacturer’s protocol. The extracted DNA from each sample (including quality control samples) was used as the template to amplify the V4 region of the 16S rRNA gene. Amplicon libraries were generated using an optimized protocol based on a previously described method (53) with slight modifications. Briefly, the V4 variable region of the 16S rRNA gene was amplified with forward and reverse primers containing common adaptor sequences and 12-bp barcodes as follows: barcode + overhang + 515 F/806R (5′-GTGCCAGCMGCCGCGGTAA-3′/5′-GGACTACHVGGGTWTCTAAT-3′) (54). PCRs were carried out using a KAPA HiFi PCR kit (KAPA Biosystems, Woburn, MA, USA). Primary PCR amplification was performed containing 2 μL diluted DNA (10 ng/μL), 1.2 μL 5× KAPA HiFi buffer, 0.18 μL 10-mM deoxynucleoside triphosphates (dNTPs), 0.3 μL dimethyl sulfoxide (DMSO; Fisher Scientific, Waltham, MA, USA), 0.12 μL KAPA HiFi polymerase, 0.3 μL for each primer (10 μM), and 0.6 μL nuclease-free water in each reaction mixture, with the following cycling conditions: 95°C for 5 min, then 20 cycles of 98°C for 20 s, 55°C for 15 s, and 72°C for 30 s, and then 72°C for 5 min. Products of the primary PCR were confirmed by 1.0% agarose gel electrophoresis, and a 1:100 dilution was used for indexing PCR with Illumina flow cell adaptors and dual indices (6 bp). Indexing PCR amplification was performed containing 5 μL 1:100 diluted product of the primary PCR, 2 μL 5× KAPA HiFi buffer, 0.3 μL 10 mM dNTPs, 0.5 μL DMSO, 0.2 μL KAPA HiFi polymerase, and 2 μL each indexing primer (5 μM), with the following cycling conditions: 95°C for 5 min, then 10 cycles of 98°C for 20 s, 55°C for 15 s, and 72°C for 1 min, and then 72°C for 5 min. AMPure XP beads (Beckman Coulter, Brea, CA, USA) were used to purify the amplicons. A Qubit double-stranded DNA (dsDNA) high-sensitivity (HS) assay kit (Invitrogen, Carlsbad, MA, USA) was used for amplicon quantification. Finally, the purified amplicons were mixed at an equal ratio for sequencing using a 2 × 250-bp Illumina MiSeq system.

Negative controls were included in each DNA extraction batch and each sequencing run. In total, we collected five “DNA extraction negative controls” that were extracted using phosphate-buffered saline (PBS) instead of samples and three “no-template negative controls” that were amplified without sample DNA. All negative controls were amplified and sequenced along with the saliva samples. Comparisons of read counts between saliva samples, DNA extraction negative controls, and no-template controls are shown in Fig. S1a. Additionally, we selected the DNA from 4 saliva samples for repeated library construction and sequencing as positive controls. We evaluated the batch-to-batch stability of the sequencing data by comparing the bacterial community of these positive controls in each batch. Principal-coordinate analysis (PCoA) plots of positive controls are shown in Fig. S1b. DNA from all saliva samples and quality control samples was extracted by the same technicians in the laboratory, following the same extraction and PCR procedures.

### 16S rRNA gene sequence analysis.

Analyses of 16S rRNA gene sequences were performed with QIIME2 2019.04 (55). Specifically, paired-end sequences were quality controlled, denoised, dereplicated, and chimera filtered by DADA2 (56) via the q2-dada2 plugin (QIIME DADA2 denoise paired) to obtain representative sequences and an amplicon sequence variant (ASV)-based feature table. Sequences were demultiplexed according to the barcodes by in-house code. Paired-end demultiplexed sequences were imported to the DADA2 pipeline with the following options: (i) truncating the forward and reverse sequences at 220 bp due to the decrease in quality, (ii) trimming 31 bp and 32 bp at the 5′ end of the forward and reverse sequences to cut the barcodes and primers reads, (iii) reads with the number of expected errors lower than two were discarded, and (iv) reads with the length of the overlap for merging the forward and reverse reads lower than 12 bp were discarded. An average of 37,430 ± 22,954 (mean ± standard deviation [SD]) sequences/sample was achieved after DADA2 filtration. ASVs present in <5 samples and with a total frequency of <5 reads were removed. Classification of 16S rRNA representative sequences was performed using the expanded Human Oral Microbiome Database (57) (version 15.2; http://www.homd.org) to reconstruct taxonomic composition. We filtered out both the case-control samples with fewer than 5,000 reads and the follow-up samples with fewer than 10,000 reads.

Alpha diversity metrics were evaluated by Shannon (taking into consideration the richness and evenness of the microbiome community), observed ASVs (the counts of unique ASVs in each sample), and Faith’s pd (taking into consideration the phylogeny of microbes to estimate diversity across a tree) indices. Beta diversity metrics were evaluated by unweighted UniFrac (phylogeny-based assessment considering the presence or absence of lineages) and weighted UniFrac (phylogeny-based assessment weighting based on the relative abundances of lineages) distance matrices with q2-diversity plugins with rarefaction to 5,000 sequences per sample (QIIME feature-table rarify).

### Statistical analysis.

Significant differences in beta diversity metrics between groups were judged using Adonis (QIIME diversity Adonis) with 999 permutations. Adonis, also known as permutational multivariate analysis of variance using distance matrices (PERMANOVA), is a function for the analysis and partitioning sums of squares using semimetric and metric distance matrices, which uses a permutation test with pseudo-*F* ratios. Significant differences in alpha diversity were judged using logistic regression models with confounders adjusted. ASV-based differential abundance comparison between patients and controls was measured by differential gene expression analysis based on the negative binomial distribution (DESeq) function (58) of the DESeq2 package (version 1.26.0) in R software. Outlier counts were filtered out based on the default Cooks distance cutoff threshold (automatic outlier filtering/replacement). An FDR-adjusted *P* value (*P*-adj value) less than 0.05 (two-sided) was considered significant.

Association analysis between the abundance of NPC-enriched taxa and serum EBV VCA-IgA levels was performed by the Spearman correlation method; three taxa with significant correlations (*P < *0.05) were shown. Individuals were classified into three groups according to whether the target taxa were detected and their median of abundance in detected individuals (low, taxa were not detected under the rarefaction depth of 5,000; medium, taxa were detected but their abundance was lower than the median of abundance in detected individuals; high, taxa were detected and their abundance was higher than the median of abundance in detected individuals). The significance between every two groups was estimated by Wilcoxon tests (two-tailed).

### Quantitative real-time PCR for Streptococcus ASV.1b51 identification.

Real-time quantitative PCR (qPCR) was performed on saliva DNA samples to identify the species information of Streptococcus ASV.1b51. Full-length reads of Streptococcus ASV.1b51 were aligned against the 16S rRNA sequence (Bacteria and Archaea) database in BLAST (https://blast.ncbi.nlm.nih.gov/Blast.cgi). Five human-isolated Streptococcus species were identified with percent identities >99%, namely, S. sanguinis, S. cristatus, S. sinensis, S. panodentis, S. parasanguinis, and S. gordonii (Table S4). These five taxa were verified by using species-specific primers. Primer sequences for the detection of each species are shown in Table S8. The amplification reactions were carried out with 1 μL template DNA (10 ng/μL), 2 μL each primer (200 μM), and 5 μL 2 × SYBR green master mix (Roche, Basel, Switzerland) in a total volume of 10 μL using the following program: 5 min at 95°C, followed by 45 cycles of 10 s at 95°C and 15 s at 55 to 60°C, according to the primer annealing temperature, and 45 s at 72°C, followed by dissociation curve generation for assessing amplicon specificity (95°C for 5 s and 65°C for 1 min and then increasing the temperature to 98°C with a rate of 0.11°C/s with continuous fluorescence detection). Meanwhile, total bacterial abundance was quantified in corresponding samples using universal bacterial primers (59) and by using the following program: 3 min at 95°C, followed by 40 cycles of 30 s at 95°C, 30 s at 53°C, and 30 s at 72°C, followed by dissociation curve generation. All reactions were performed with two technical replicates on a Roche LightCycler 480 II instrument. The relative abundance of these assumed species was calculated by the threshold cycle (2^−ΔΔ^*^CT^*) method (Δ*C_T_* = average *C_T_* values of Streptococcus species − average *C_T_* values of total bacteria).

### EBV antibody tests.

Five microliters of each blood sample was collected for serum EBV VCA-IgA antibody tests. The serum VCA-IgA antibody levels of EBV were measured by using commercial enzyme-linked immunosorbent assay kits (Zhongshan Biotech, Zhongshan, China) as described previously (32, 60). All tests were conducted by the same technicians at Sun Yat-Sen University Cancer Center. Optical density (OD) values were standardized by calculating the ratio of the optical density of the sample over that of the reference control.

### EBV quantification by real-time qPCR.

EBV quantification was performed as described previously (61). The RT-PCR consisted of the amplification primers BW-F, 5′-CCCAACACTCCACCACACC-3′, and BW-R, 5′-TCTTAGGAGCTGTCCGAGGG-3′, and a dually labeled fluorescent TaqMan probe, BW-probe, 5′-(FAM) CACACACTACACACACCCACCCGTCTC (TAMRA)-3′. Each PCR was performed containing 4 μL LightCycler 480 probes master mix (Roche, Basel, Switzerland), 1 μL each primer, 0.2 μL probe, 0.8 μL water, and 2 μL DNA template and was performed on a Roche LightCycler 480. Samples were tested in duplicate. The PCR conditions were as follows: 5 min at 95°C, followed by 45 cycles of denaturation for 30 s at 95°C, annealing for 30 s at 60°C, and extension for 15 s at 72°C. Standard sample ladders, which contained the BamHI-W region of the EBV genome (0, 10^2^, 10^3^, 10^4^, 10^5^, 10^6^, and 10^7^ copies per 2 μL), were used to draw a standard curve.

### Bacterial strain and cell line.

Streptococcus sanguinis (CCTCC AB99004 or ATCC 10556) was obtained from the China Center for Type Culture Collection. S. sanguinis was maintained in brain heart infusion broth (BHI; BD, Franklin Lakes, NJ, USA) at 37°C and 175 rpm. Streptococcus salivarius (GDMCC 1.949 or ATCC 13419) was obtained from the Guangdong Microbial Culture Collection Center, China. S. salivarius was maintained in tryptic soy broth (BD, Franklin Lakes, NJ, USA) at 37°C and 175 rpm. The EBV-positive Akata cell line was purchased from Jennio Biotech (Guangzhou, Guangdong, China), cultured in RPMI 1640 medium supplemented with 10% fetal bovine serum (FBS), and maintained in a humidified incubator containing 5% CO_2_ at 37°C. This Akata cell line carried a modified EBV genome with coexpression of GFP, which allowed us to determine the positivity of EBV using inverted fluorescence microscopy or flow cytometry.

### Coculture experiments of Streptococcus spp. and Akata cells.

For absolute bacterial quantification, S. sanguinis was maintained in brain heart infusion broth with 5% CO_2_ at 37°C for 48 h. S. salivarius was maintained in tryptic soy broth with 5% CO_2_ at 37°C for 48 h. The absorbance of the bacterial solutions was detected using a microplate reader (Bio-Tek Epoch 2) at 600 nm. The bacterial solutions were then calibrated to OD_600_ values of 0.6, 0.5, 0.4, 0.3, and 0.2 as standard sample ladders. Next, standard sample ladders were diluted 10,000 times. Fifty microliters from each dilution was added onto BHI agar plates and cultured at 37°C and 5% CO_2_. After 48 h of culturing, the number of colonies on each plate was counted. Then, we calculated the number of cells per milliliter of original standard sample ladders. Finally, the number of cells was combined with the corresponding OD_600_ value for each standard sample to generate the standard curves for S. sanguinis and S. salivarius.

Akata cells were seeded in a 12-well plate at 2 × 10^5^ cells per well. The cells were treated with 15 μM H_2_O_2_ or cocultured with different amounts of S. sanguinis (multiplicity of infection [MOI] = 10, 20, 40), PBS, and IgG (0.8%) were used as the mock control and positive control, respectively. Catalase was added to the culture system at a final concentration of 200 units/mL to eliminate H_2_O_2_. The cells were harvested and resuspended in PBS after 24 h of incubation and then subjected to flow cytometry to analyze the EBV-positive rate of the cells.

### H_2_O_2_ concentration detection.

The H_2_O_2_ concentration was detected by a hydrogen peroxide assay kit (Abcam, Cambridge, MA, USA) using a fluorometric method. Coculture samples, saliva, and bacterial culture supernatant were centrifuged at 3,000 × *g* for 5 min, and the supernatant was obtained to detect H_2_O_2_ concentration following the manufacturers’ instructions. Fluorescence was measured on a microplate reader (Tecan Spark) with red fluorescence (excitation and emission, 535 and 587 nm, respectively). All experiments were performed in duplicate.

### RNA sequencing and data analysis.

Akata cells treated with H_2_O_2_ or cocultured with S. sanguinis for 24 h were harvested and resuspended in TRIzol. RNA was extracted by a HiPure universal RNA minikit (Magen Tech, Guangzhou, China) according to the manufacturer’s protocol. rRNA was removed from the total RNA using the NEBNext rRNA depletion kit (New England Biolabs, Beverly, MA, USA) before library construction. The lncRNA library was constructed by the NEBNext Ultra Directional RNA library prep kit (New England Biolabs, Beverly, MA, USA) and sequenced using a NovaSeq6000 PE150 sequencer, generating an average of 12 G data per sample.

Adaptors were trimmed from the RNA-seq reads, and Trimmomatic-0.38 (62) was used for quality control. The qualified reads were aligned to the human reference genome (hg38; Genome Reference Consortium GRCH38) and a modified Akata-EBV genome using HISAT2 (63). An expression matrix of gene counts was generated using HTseq-count using the default parameters (64). Genes differentially expressed between treatment samples and blank group samples were measured based on the Empirical Analysis of Digital Gene Expression Data in R (edgeR) package (65) (version 3.30.3). Raw read counts were used as the input for the edgeR analysis. Statistically significant gene sets were identified using gene set enrichment analysis (GSEA) software (66) (version 4.1.0). Gene expression counts normalized by the TMM method were used as the input for GSEA. We chose hallmark gene sets (version 7.2) as the gene set database and analyzed them with 1,000,000 permutations.

### EBV gene expression quantitation by real-time qPCR.

Akata cells cocultured with S. sanguinis for 24 h were harvested and resuspended in TRIzol. RNA was extracted by a HiPure universal RNA minikit (Magen Tech, Guangzhou, China) according to the manufacturer’s protocol. RNA was reverse transcribed to cDNA by a PrimeScript reverse transcription (RT) reagent kit with gDNA Eraser (TaKaRa, Tokyo, Japan) prior to qPCR. The EBV genes *BRLF1*, *BMLF1*, *BLLF1*, *BZLF2*, *BXLF2*, *BKRF2*, *BALF4*, *LMP1*, *LMP2A*, and *EBNA1* and the host gene, GAPDH (glyceraldehyde-3-phosphate dehydrogenase), were quantified. The amplification primers corresponding to these genes are shown in Table S9. Each PCR was performed containing 4 μL LightCycler 480 SYBR green I master mix (Roche, Basel, Switzerland), 1 μL each primer, 2 μL water, and 1 μL cDNA template and was performed on a Roche LightCycler 480. Samples were tested in duplicate. PCRs were performed with the following conditions: 5 min at 95°C, followed by 45 cycles of denaturation for 10 s at 95°C, annealing for 20 s at 58°C, and extension for 20 s at 72°C. The relative abundance of these assumed species was calculated by the 2^−ΔΔ^*^CT^* method (Δ*C_T_* = *C_T_* values of EBV target genes − *C_T_* values of GAPDH).

### EBV methylation sequencing and data analysis.

Akata cells cocultured with S. sanguinis for 24 h were harvested, and the DNA was extracted for EBV methylation sequencing. We sequenced three coculture samples, blank, S. sanguinis MOI of 20 treatment, and S. sanguinis MOI of 40 treatment. The DNA was fragmented, and a genomic library was constructed using the VariantBaits target enrichment library prep kit (LC-Bio Tech, Hangzhou, China). The genomic library was hybridized with the EBV probe to specifically capture the EBV sequence. The captured library was treated with bisulfite conversion using the Zymo Research EZ DNA Methylation-Gold kit (Zymo Research, CA, USA). Next, the bisulfite-converted library was amplified by indexing PCR. The libraries were sequenced using NovaSeq6000 PE150. The sequencing data were analyzed using the Methy-Pipe2 pipeline (67). The paired-end sequencing data were aligned to the *in silico*-converted reference genomes containing a human genome (hg38; Genome Reference Consortium GRCH38) and a modified Akata-EBV genome that was C-to-T converted. CpG methylation density was defined as the number of C nucleotides minus the total number of C and T nucleotides that overlapped with each genomic cytosine site across the region.

### Data availability.

The data sets supporting the conclusions of this article are available in the National Center for Biotechnology Information (NCBI) Sequence Read Archive (SRA) under BioProject numbers PRJNA721474 and PRJNA721325 for 16S rRNA sequencing data (including sequence data of experimental controls), PRJNA695400 for RNA-seq, and PRJNA695112 for EBV methylation sequencing. The patient information and related data sets have also been deposited in the Research Data Deposit public platform (www.researchdata.org.cn, accession number RDDA2021001990). The data sets supporting the conclusions of this article are included in this article and its supplementary files.

## References

[1] Bray F, Ferlay J, Soerjomataram I, Siegel RL, Torre LA, Jemal A. 2018. Global cancer statistics 2018: GLOBOCAN estimates of incidence and mortality worldwide for 36 cancers in 185 countries. CA Cancer J Clin 68:394–424. doi:10.3322/caac.21492. 30207593

[2] Chen Y-P, Chan ATC, Le Q-T, Blanchard P, Sun Y, Ma J. 2019. Nasopharyngeal carcinoma. Lancet 394:64–80. doi:10.1016/s0140-6736(19)30956-0. 31178151

[3] Young LS, Yap LF, Murray PG. 2016. Epstein-Barr virus: more than 50 years old and still providing surprises. Nat Rev Cancer 16:789–802. doi:10.1038/nrc.2016.92. 27687982

[4] IARC Working Group on the Evaluation of Carcinogenic Risks to Humans. 2012. Biological agents. Volume 100 B. A review of human carcinogens. IARC Monogr Eval Carcinog Risks Hum 100:1–441. PMC4781184 23189750

[5] Wong Y, Meehan MT, Burrows SR, Doolan DL, Miles JJ. 2022. Estimating the global burden of Epstein–Barr virus-related cancers. J Cancer Res Clin Oncol 148:31–46. doi:10.1007/s00432-021-03824-y. 34705104 PMC8752571

[6] de Martel C, Georges D, Bray F, Ferlay J, Clifford GM. 2020. Global burden of cancer attributable to infections in 2018: a worldwide incidence analysis. Lancet Glob Health 8:e180–e190. doi:10.1016/S2214-109X(19)30488-7. 31862245

[7] He Y-Q, Liao X-Y, Xue W-Q, Xu Y-F, Xu F-H, Li F-F, Li X-Z, Zhang J-B, Wang T-M, Wang F, Yu H-L, Feng Q-S, Chen L-Z, Cao S-M, Liu Q, Mu J, Jia W-H. 2019. Association between environmental factors and oral Epstein-Barr virus DNA loads: a multicenter cross-sectional study in China. J Infect Dis 219:400–409. doi:10.1093/infdis/jiy542. 30307559 PMC6941616

[8] Flemer B, Warren RD, Barrett MP, Cisek K, Das A, Jeffery IB, Hurley E, O'Riordain M, Shanahan F, O'Toole PW. 2018. The oral microbiota in colorectal cancer is distinctive and predictive. Gut 67:1454–1463. doi:10.1136/gutjnl-2017-314814. 28988196 PMC6204958

[9] Fan X, Alekseyenko AV, Wu J, Peters BA, Jacobs EJ, Gapstur SM, Purdue MP, Abnet CC, Stolzenberg-Solomon R, Miller G, Ravel J, Hayes RB, Ahn J. 2018. Human oral microbiome and prospective risk for pancreatic cancer: a population-based nested case-control study. Gut 67:120–127. doi:10.1136/gutjnl-2016-312580. 27742762 PMC5607064

[10] Hashim D, Sartori S, Brennan P, Curado MP, Wünsch-Filho V, Divaris K, Olshan AF, Zevallos JP, Winn DM, Franceschi S, Castellsagué X, Lissowska J, Rudnai P, Matsuo K, Morgenstern H, Chen C, Vaughan TL, Hofmann JN, D'Souza G, Haddad RI, Wu H, Lee Y-C, Hashibe M, Vecchia CL, Boffetta P. 2016. The role of oral hygiene in head and neck cancer: results from International Head and Neck Cancer Epidemiology (INHANCE) consortium. Ann Oncol 27:1619–1625. doi:10.1093/annonc/mdw224. 27234641 PMC4959929

[11] Liu Z, Chang ET, Liu Q, Cai Y, Zhang Z, Chen G, Xie S-H, Cao S-M, Shao J-Y, Jia W-H, Zheng Y, Liao J, Chen Y, Ernberg I, Vaughan TL, Adami H-O, Huang G, Zeng Y, Zeng Y-X, Ye W. 2016. Oral hygiene and risk of nasopharyngeal carcinoma—a population-based case–control study in China. Cancer Epidemiol Biomarkers Prev 25:1201–1207. doi:10.1158/1055-9965.EPI-16-0149. 27197279 PMC4970945

[12] Huang J, Roosaar A, Axéll T, Ye W. 2016. A prospective cohort study on poor oral hygiene and pancreatic cancer risk. Int J Cancer 138:340–347. doi:10.1002/ijc.29710. 26235255

[13] Helmink BA, Khan MAW, Hermann A, Gopalakrishnan V, Wargo JA. 2019. The microbiome, cancer, and cancer therapy. Nat Med 25:377–388. doi:10.1038/s41591-019-0377-7. 30842679

[14] Schwabe RF, Jobin C. 2013. The microbiome and cancer. Nat Rev Cancer 13:800–812. doi:10.1038/nrc3610. 24132111 PMC3986062

[15] Coker OO, Dai Z, Nie Y, Zhao G, Cao L, Nakatsu G, Wu WK, Wong SH, Chen Z, Sung JJY, Yu J. 2018. Mucosal microbiome dysbiosis in gastric carcinogenesis. Gut 67:1024–1032. doi:10.1136/gutjnl-2017-314281. 28765474 PMC5969346

[16] Yu J, Feng Q, Wong SH, Zhang D, Liang QY, Qin Y, Tang L, Zhao H, Stenvang J, Li Y, Wang X, Xu X, Chen N, Wu WKK, Al-Aama J, Nielsen HJ, Kiilerich P, Jensen BAH, Yau TO, Lan Z, Jia H, Li J, Xiao L, Lam TYT, Ng SC, Cheng AS-L, Wong VW-S, Chan FKL, Xu X, Yang H, Madsen L, Datz C, Tilg H, Wang J, Brünner N, Kristiansen K, Arumugam M, Sung JJ-Y, Wang J. 2017. Metagenomic analysis of faecal microbiome as a tool towards targeted non-invasive biomarkers for colorectal cancer. Gut 66:70–78. doi:10.1136/gutjnl-2015-309800. 26408641

[17] He Y-Q, Zhou T, Yang D-W, Jia Y-J, Yuan L-L, Zhang W-L, Wang T-M, Liao Y, Xue W-Q, Zhang J-B, Zheng X-H, Li X-Z, Zhang P-F, Zhang S-D, Hu Y-Z, Wang F, Cho WC, Ma J, Sun Y, Jia W-H. 2021. Prognostic value of oral Epstein–Barr virus DNA load in locoregionally advanced nasopharyngeal carcinoma. Front Mol Biosci 8:757644. doi:10.3389/fmolb.2021.757644. 35096963 PMC8793774

[18] Zhu X-X, Yang X-J, Chao Y-L, Zheng H-M, Sheng H-F, Liu H-Y, He Y, Zhou H-W. 2017. The potential effect of oral microbiota in the prediction of mucositis during radiotherapy for nasopharyngeal carcinoma. EBioMedicine 18:23–31. doi:10.1016/j.ebiom.2017.02.002. 28216066 PMC5405060

[19] Du J-L, Chen S-H, Huang Q-H, Xie S-H, Ye Y-F, Gao R, Guo J, Yang M-J, Liu Q, Hong M-H, Cao S-M. 2016. Subtype distribution and long-term titer fluctuation patterns of serum anti-Epstein-Barr virus antibodies in a non-nasopharyngeal carcinoma population from an endemic area in South China: a cohort study. Chin J Cancer 35:78. doi:10.1186/s40880-016-0130-2. 27527073 PMC4986177

[20] Chen H, Chi P, Wang W, Li L, Luo Y, Fu J, Zhang L, Liu W. 2014. Evaluation of a semi-quantitative ELISA for IgA antibody against Epstein-Barr virus capsid antigen in the serological diagnosis of nasopharyngeal carcinoma. Int J Infect Dis 25:110–115. doi:10.1016/j.ijid.2014.03.1373. 24878579

[21] Kanda T, Yajima M, Ahsan N, Tanaka M, Takada K. 2004. Production of high-titer Epstein-Barr virus recombinants derived from Akata cells by using a bacterial artificial chromosome system. J Virol 78:7004–7015. doi:10.1128/JVI.78.13.7004-7015.2004. 15194777 PMC421639

[22] Zhu L, Kreth J. 2012. The role of hydrogen peroxide in environmental adaptation of oral microbial communities. Oxid Med Cell Longev 2012:717843. doi:10.1155/2012/717843. 22848782 PMC3405655

[23] Cerimele F, Battle T, Lynch R, Frank DA, Murad E, Cohen C, Macaron N, Sixbey J, Smith K, Watnick RS, Eliopoulos A, Shehata B, Arbiser JL. 2005. Reactive oxygen signaling and MAPK activation distinguish Epstein-Barr Virus (EBV)-positive versus EBV-negative Burkitt’s lymphoma. Proc Natl Acad Sci USA 102:175–179. doi:10.1073/pnas.0408381102. 15611471 PMC544042

[24] O'Grady T, Cao S, Strong MJ, Concha M, Wang X, Splinter Bondurant S, Adams M, Baddoo M, Srivastav SK, Lin Z, Fewell C, Yin Q, Flemington EK. 2014. Global bidirectional transcription of the Epstein-Barr virus genome during reactivation. J Virol 88:1604–1616. doi:10.1128/JVI.02989-13. 24257595 PMC3911580

[25] Debelius JW, Huang T, Cai Y, Ploner A, Barrett D, Zhou X, Xiao X, Li Y, Liao J, Zheng Y, Huang G, Adami H-O, Zeng Y, Zhang Z, Ye W. 2020. Subspecies niche specialization in the oral microbiome is associated with nasopharyngeal carcinoma risk. mSystems 5:e00065-20. doi:10.1128/mSystems.00065-20. 32636333 PMC7343305

[26] Du Y, Feng R, Chang ET, Debelius JW, Yin L, Xu M, Huang T, Zhou X, Xiao X, Li Y, Liao J, Zheng Y, Huang G, Adami H-O, Zhang Z, Cai Y, Ye W. 2022. Influence of pre-treatment saliva microbial diversity and composition on nasopharyngeal carcinoma prognosis. Front Cell Infect Microbiol 12:831409. doi:10.3389/fcimb.2022.831409. 35392614 PMC8981580

[27] Hou J, Zheng H, Li P, Liu H, Zhou H, Yang X. 2018. Distinct shifts in the oral microbiota are associated with the progression and aggravation of mucositis during radiotherapy. Radiother Oncol 129:44–51. doi:10.1016/j.radonc.2018.04.023. 29735410

[28] McKenzie J, El-Guindy A. 2015. Epstein-Barr virus lytic cycle reactivation. Curr Top Microbiol Immunol 391:237–261. doi:10.1007/978-3-319-22834-1_8. 26428377

[29] Li H, Liu S, Hu J, Luo X, Li N, M Bode A, Cao Y. 2016. Epstein-Barr virus lytic reactivation regulation and its pathogenic role in carcinogenesis. Int J Biol Sci 12:1309–1318. doi:10.7150/ijbs.16564. 27877083 PMC5118777

[30] Münz C. 2019. Latency and lytic replication in Epstein–Barr virus-associated oncogenesis. Nat Rev Microbiol 17:691–700. doi:10.1038/s41579-019-0249-7. 31477887

[31] Ji MF, Wang DK, Yu YL, Guo YQ, Liang JS, Cheng WM, Zong YS, Chan KH, Ng SP, Wei WI, Chua DTT, Sham JST, Ng MH. 2007. Sustained elevation of Epstein–Barr virus antibody levels preceding clinical onset of nasopharyngeal carcinoma. Br J Cancer 96:623–630. doi:10.1038/sj.bjc.6603609. 17285127 PMC2360049

[32] Xu F-H, Xiong D, Xu Y-F, Cao S-M, Xue W-Q, Qin H-D, Liu W-S, Cao J-Y, Zhang Y, Feng Q-S, Chen L-Z, Li M-Z, Liu Z-W, Liu Q, Hong M-H, Shugart YY, Zeng Y-X, Zeng M-S, Jia W-H. 2012. An epidemiological and molecular study of the relationship between smoking, risk of nasopharyngeal carcinoma, and Epstein–Barr virus activation. J Natl Cancer Inst 104:1396–1410. doi:10.1093/jnci/djs320. 22972969

[33] Frisan T, Nagy N, Chioureas D, Terol M, Grasso F, Masucci MG. 2019. A bacterial genotoxin causes virus reactivation and genomic instability in Epstein–Barr virus infected epithelial cells pointing to a role of co-infection in viral oncogenesis. Int J Cancer 144:98–109. doi:10.1002/ijc.31652. 29978480 PMC6587852

[34] Makino K, Takeichi O, Imai K, Inoue H, Hatori K, Himi K, Saito I, Ochiai K, Ogiso B. 2018. Porphyromonas endodontalis reactivates latent Epstein–Barr virus. Int Endod J 51:1410–1419. doi:10.1111/iej.12959. 29858508

[35] Sakamoto H, Naito H, Ohta Y, Tanakna R, Maeda N, Sasaki J, Nord CE. 1999. Isolation of bacteria from cervical lymph nodes in patients with oral cancer. Arch Oral Biol 44:789–793. doi:10.1016/s0003-9969(99)00079-5. 10530911

[36] Khera AV, Chaffin M, Aragam KG, Haas ME, Roselli C, Choi SH, Natarajan P, Lander ES, Lubitz SA, Ellinor PT, Kathiresan S. 2018. Genome-wide polygenic scores for common diseases identify individuals with risk equivalent to monogenic mutations. Nat Genet 50:1219–1224. doi:10.1038/s41588-018-0183-z. 30104762 PMC6128408

[37] Oya Y, Tonomura A, Yamamoto K. 1987. The biological activity of hydrogen peroxide. III. Induction of Epstein-Barr virus via indirect action, as compared with TPA and teleocidin. Int J Cancer 40:69–73. doi:10.1002/ijc.2910400113. 3036721

[38] Gloire G, Legrand-Poels S, Piette J. 2006. NF-kappaB activation by reactive oxygen species: fifteen years later. Biochem Pharmacol 72:1493–1505. doi:10.1016/j.bcp.2006.04.011. 16723122

[39] Chung GT-Y, Lou WP-K, Chow C, To K-F, Choy K-W, Leung AW-C, Tong CY-K, Yuen JW-F, Ko C-W, Yip TT-C, Busson P, Lo K-W. 2013. Constitutive activation of distinct NF-κB signals in EBV-associated nasopharyngeal carcinoma. J Pathol 231:311–322. doi:10.1002/path.4239. 23868181

[40] Keller SA, Hernandez-Hopkins D, Vider J, Ponomarev V, Hyjek E, Schattner EJ, Cesarman E. 2006. NF-kappaB is essential for the progression of KSHV- and EBV-infected lymphomas in vivo. Blood 107:3295–3302. doi:10.1182/blood-2005-07-2730. 16380446 PMC1432097

[41] Long X, Wong CC, Tong L, Chu ESH, Ho Szeto C, Go MYY, Coker OO, Chan AWH, Chan FKL, Sung JJY, Yu J. 2019. Peptostreptococcus anaerobius promotes colorectal carcinogenesis and modulates tumour immunity. Nat Microbiol 4:2319–2330. doi:10.1038/s41564-019-0541-3. 31501538

[42] Zhang L, Liu Y, Zheng HJ, Zhang CP. 2019. The oral microbiota may have influence on oral cancer. Front Cell Infect Microbiol 9:476. doi:10.3389/fcimb.2019.00476. 32010645 PMC6974454

[43] Murad CF, Sassone LM, Faveri M, Hirata R, Figueiredo L, Feres M. 2014. Microbial diversity in persistent root canal infections investigated by checkerboard DNA-DNA hybridization. J Endod 40:899–906. doi:10.1016/j.joen.2014.02.010. 24935532

[44] Ganly I, Yang L, Giese RA, Hao Y, Nossa CW, Morris LGT, Rosenthal M, Migliacci J, Kelly D, Tseng W, Hu J, Li H, Brown S, Pei Z. 2019. Periodontal pathogens are a risk factor of oral cavity squamous cell carcinoma, independent of tobacco and alcohol and human papillomavirus. Int J Cancer 145:775–784. doi:10.1002/ijc.32152. 30671943 PMC6554043

[45] Chipashvili O, Utter DR, Bedree JK, Ma Y, Schulte F, Mascarin G, Alayyoubi Y, Chouhan D, Hardt M, Bidlack F, Hasturk H, He X, McLean JS, Bor B. 2021. Episymbiotic Saccharibacteria suppresses gingival inflammation and bone loss in mice through host bacterial modulation. Cell Host Microbe 29:1649–1662.e1647. doi:10.1016/j.chom.2021.09.009. 34637779 PMC8595704

[46] Shi J, Yang Y, Xie H, Wang X, Wu J, Long J, Courtney R, Shu X-O, Zheng W, Blot WJ, Cai Q. 2021. Association of oral microbiota with lung cancer risk in a low-income population in the Southeastern USA. Cancer Causes Control 32:1423–1432. doi:10.1007/s10552-021-01490-6. 34432217 PMC8541916

[47] Duan X-B, Wu T-X, Guo Y-C, Zhou X-D, Lei Y-L, Xu X, Mo A-C, Wang Y-Y, Yuan Q. 2017. Marginal bone loss around non-submerged implants is associated with salivary microbiome during bone healing. Int J Oral Sci 9:95–103. doi:10.1038/ijos.2017.18. 28621324 PMC5518974

[48] Man WH, Clerc M, de Steenhuijsen Piters WAA, van Houten MA, Chu MLJN, Kool J, Keijser BJF, Sanders EAM, Bogaert D. 2019. Loss of microbial topography between oral and nasopharyngeal microbiota and development of respiratory infections early in life. Am J Respir Crit Care Med 200:760–770. doi:10.1164/rccm.201810-1993OC. 30883192

[49] Roy S, Trinchieri G. 2017. Microbiota: a key orchestrator of cancer therapy. Nat Rev Cancer 17:271–285. doi:10.1038/nrc.2017.13. 28303904

[50] Cheng WY, Wu CY, Yu J. 2020. The role of gut microbiota in cancer treatment: friend or foe? Gut 69:1867–1876. doi:10.1136/gutjnl-2020-321153. 32759302 PMC7497589

[51] Montassier E, Al-Ghalith GA, Hillmann B, Viskocil K, Kabage AJ, McKinlay CE, Sadowsky MJ, Khoruts A, Knights D. 2018. CLOUD: a non-parametric detection test for microbiome outliers. Microbiome 6:137. doi:10.1186/s40168-018-0514-4. 30081949 PMC6080375

[52] Jia Y-J, Liao Y, He Y-Q, Zheng M-Q, Tong X-T, Xue W-Q, Zhang J-B, Yuan L-L, Zhang W-L, Jia W-H. 2021. Association between oral microbiota and cigarette smoking in the Chinese population. Front Cell Infect Microbiol 11:658203. doi:10.3389/fcimb.2021.658203. 34123872 PMC8195269

[53] Gohl DM, Vangay P, Garbe J, MacLean A, Hauge A, Becker A, Gould TJ, Clayton JB, Johnson TJ, Hunter R, Knights D, Beckman KB. 2016. Systematic improvement of amplicon marker gene methods for increased accuracy in microbiome studies. Nat Biotechnol 34:942–949. doi:10.1038/nbt.3601. 27454739

[54] Caporaso JG, Lauber CL, Walters WA, Berg-Lyons D, Lozupone CA, Turnbaugh PJ, Fierer N, Knight R. 2011. Global patterns of 16S rRNA diversity at a depth of millions of sequences per sample. Proc Natl Acad Sci USA 108(Suppl 1):4516–4522. doi:10.1073/pnas.1000080107. 20534432 PMC3063599

[55] Bolyen E, Rideout JR, Dillon MR, Bokulich NA, Abnet CC, Al-Ghalith GA, Alexander H, Alm EJ, Arumugam M, Asnicar F, Bai Y, Bisanz JE, Bittinger K, Brejnrod A, Brislawn CJ, Brown CT, Callahan BJ, Caraballo-Rodríguez AM, Chase J, Cope EK, Da Silva R, Diener C, Dorrestein PC, Douglas GM, Durall DM, Duvallet C, Edwardson CF, Ernst M, Estaki M, Fouquier J, Gauglitz JM, Gibbons SM, Gibson DL, Gonzalez A, Gorlick K, Guo J, Hillmann B, Holmes S, Holste H, Huttenhower C, Huttley GA, Janssen S, Jarmusch AK, Jiang L, Kaehler BD, Kang KB, Keefe CR, Keim P, Kelley ST, Knights D. 2019. Reproducible, interactive, scalable and extensible microbiome data science using QIIME 2. Nat Biotechnol 37:852–857. doi:10.1038/s41587-019-0209-9. 31341288 PMC7015180

[56] Callahan BJ, McMurdie PJ, Rosen MJ, Han AW, Johnson AJA, Holmes SP. 2016. DADA2: high-resolution sample inference from Illumina amplicon data. Nat Methods 13:581–583. doi:10.1038/nmeth.3869. 27214047 PMC4927377

[57] Escapa IF, Chen T, Huang Y, Gajare P, Dewhirst FE, Lemon KP. 2018. New insights into human nostril microbiome from the Expanded Human Oral Microbiome Database (eHOMD): a resource for the microbiome of the human aerodigestive tract. mSystems 3:e00187-18. doi:10.1128/mSystems.00187-18. PMC6280432 30534599

[58] Love MI, Huber W, Anders S. 2014. Moderated estimation of fold change and dispersion for RNA-seq data with DESeq2. Genome Biol 15:550. doi:10.1186/s13059-014-0550-8. 25516281 PMC4302049

[59] Wong SH, Kwong TNY, Chow T-C, Luk AKC, Dai RZW, Nakatsu G, Lam TYT, Zhang L, Wu JCY, Chan FKL, Ng SSM, Wong MCS, Ng SC, Wu WKK, Yu J, Sung JJY. 2017. Quantitation of faecal Fusobacterium improves faecal immunochemical test in detecting advanced colorectal neoplasia. Gut 66:1441–1448. doi:10.1136/gutjnl-2016-312766. 27797940 PMC5530471

[60] He Y-Q, Xue W-Q, Xu F-H, Xu Y-F, Zhang J-B, Yu H-L, Feng Q-S, Chen L-Z, Cao S-M, Liu Q, Mu J, Zeng Y-X, Jia W-H. 2018. The relationship between environmental factors and the profile of Epstein-Barr virus antibodies in the lytic and latent infection periods in healthy populations from endemic and non-endemic nasopharyngeal carcinoma areas in China. EBioMedicine 30:184–191. doi:10.1016/j.ebiom.2018.02.019. 29606628 PMC5952216

[61] Zheng XH, Lu LX, Li XZ, Jia WH. 2015. Quantification of Epstein–Barr virus DNA load in nasopharyngeal brushing samples in the diagnosis of nasopharyngeal carcinoma in southern China. Cancer Sci 106:1196–1201. doi:10.1111/cas.12718. 26082292 PMC4582989

[62] Bolger AM, Lohse M, Usadel B. 2014. Trimmomatic: a flexible trimmer for Illumina sequence data. Bioinformatics 30:2114–2120. doi:10.1093/bioinformatics/btu170. 24695404 PMC4103590

[63] Kim D, Langmead B, Salzberg SL. 2015. HISAT: a fast spliced aligner with low memory requirements. Nat Methods 12:357–360. doi:10.1038/nmeth.3317. 25751142 PMC4655817

[64] Anders S, Pyl PT, Huber W. 2015. HTSeq—a Python framework to work with high-throughput sequencing data. Bioinformatics 31:166–169. doi:10.1093/bioinformatics/btu638. 25260700 PMC4287950

[65] Robinson MD, McCarthy DJ, Smyth GK. 2010. edgeR: a Bioconductor package for differential expression analysis of digital gene expression data. Bioinformatics 26:139–140. doi:10.1093/bioinformatics/btp616. 19910308 PMC2796818

[66] Subramanian A, Tamayo P, Mootha VK, Mukherjee S, Ebert BL, Gillette MA, Paulovich A, Pomeroy SL, Golub TR, Lander ES, Mesirov JP. 2005. Gene set enrichment analysis: a knowledge-based approach for interpreting genome-wide expression profiles. Proc Natl Acad Sci USA 102:15545–15550. doi:10.1073/pnas.0506580102. 16199517 PMC1239896

[67] Jiang P, Sun K, Lun FMF, Guo AM, Wang H, Chan KCA, Chiu RWK, Lo YMD, Sun H. 2014. Methy-Pipe: an integrated bioinformatics pipeline for whole genome bisulfite sequencing data analysis. PLoS One 9:e100360. doi:10.1371/journal.pone.0100360. 24945300 PMC4063866

