# Supplementary material for: Oral Microbiota Alteration and Roles in Epstein-Barr Virus Reactivation in Nasopharyngeal Carcinoma
Source: Microbiol Spectr. 2023 Jan 16;11(1):e03448-22. doi: 10.1128/spectrum.03448-22 (PMC9927204; doi:10.1128/spectrum.03448-22)
Supplement: Supplemental file 1 — Supplemental material. Download spectrum.03448-22-s0001.pdf, PDF file, 1.4 MB [file spectrum.03448-22-s0001.pdf]

## Appendix 1. Supplementary Figures

**Supplementary Figure 1.** Summary of the quality control of 16S rRNA sequencing in this study.

**Supplementary Figure 2.** Scatter plot showing the correlation between the relative abundance of *Streptococcus*_ASV.1b51 obtained from 16S rRNA sequencing and the relative abundance obtained from qPCR with *S. sanguinis*-specific primers.

**Supplementary Figure 3.** The association between the abundance of NPC-enriched taxa and serum EBV VCA-IgA levels.

**Supplementary Figure 4.** $H_2O_2$ concentration of the culture supernatant of *S. sanguinis* after 24 hours of culture.

**Supplementary Figure 5.** The $H_2O_2$ concentration in coculture supernatant with different culture times.

**Supplementary Figure 6.** Independent qPCR validation of EBV genes expression of $H_2O_2$ treated and *S.sanguinis* cocultured samples.

**Supplementary Figure 7.** Heatmap of methylation rates of EBV encoded genes in untreated and *S.sanguinis* cocultured Akata cells.

## Appendix 2. Supplementary Tables

**Supplementary Table 1.** The demographic characteristics of participants in this study.

**Supplementary Table 2.** Alpha and beta diversity analysis of oral microbiota and environmental factors.

**Supplementary Table 3.** The relative abundance difference of oral taxa between NPC patients and controls.

**Supplementary Table 4.** Sequence alignments of *Streptococcus* species with read of *Streptococcus*_ASV.1b51.

**Supplementary Table 5.** Enriched hallmark pathways and core enrichment genes of RNA-seq.

**Supplementary Table 6.** Differential expression genes of EBV in coculture samples.

**Supplementary Table 7.** EBV genes methylation rates in coculture samples.

**Supplementary Table 8.** Primers for identifying the species of *Streptococcus*_ASV.1b51.

**Supplementary Table 9.** EBV specific primers and probes used in this study.

## Appendix 1. Supplementary Figures

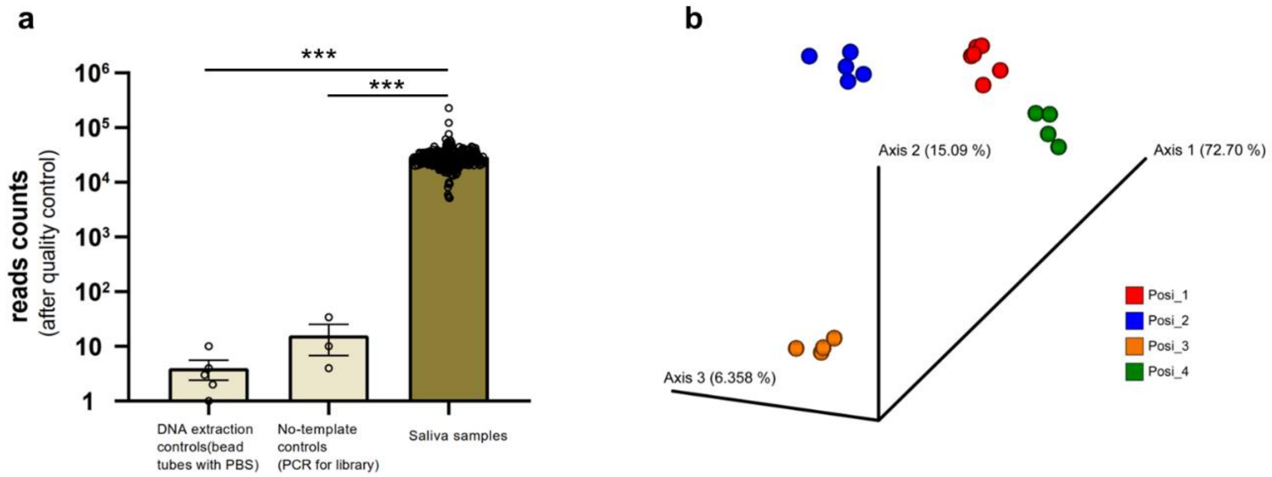


**Supplementary Figure 1: Summary of the quality control of 16S rRNA sequencing in this study.** **a** Reads counts of negative controls, no-template negative controls and saliva samples. The read counts of 16S rRNA sequencing were significantly lower in negative controls compared with saliva samples. **b** PCoA plot of Weighted Unifrac distance of four positive controls. Saliva sample DNAs from four individuals were chosen as positive controls and were amplified and sequenced in each sequencing batch. Weighted Unifrac was used to generate distance between all positive controls and were displayed using PCoA. Points were grouped in four clusters according to donated individuals.

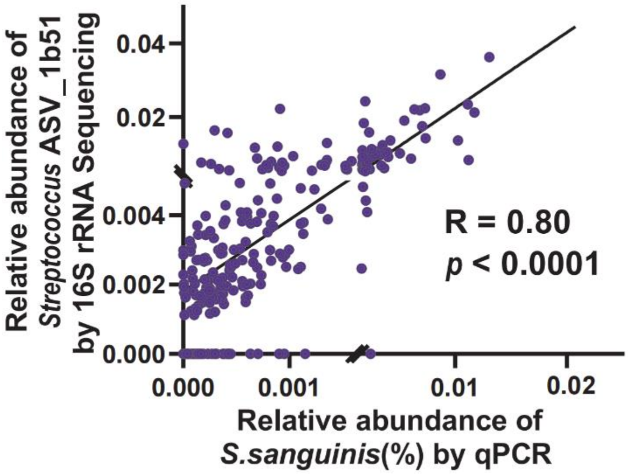


**Supplementary Figure2: Scatter plot showing the correlation between the relative abundance of *Streptococcus*_ASV.1b51 obtained from 16S rRNA sequencing and the relative abundance obtained from qPCR with *S. sanguinis*-specific primers.** Correlation was tested by Spearman analysis

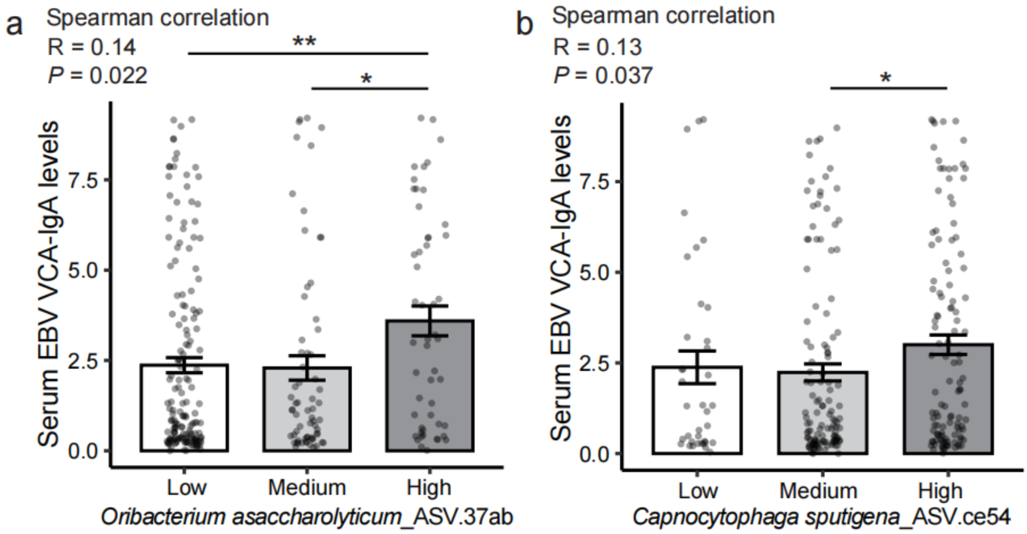


**Supplementary Figure3: the association between the abundance of *O.asaccharolyticum* (a) and *C.sputigena* (b) and serum EBV VCA-IgA levels.** Individuals were classified to three groups according to whether detected the target taxa and its median of abundance in detected individuals (Low: taxa were not detected under the rarefaction depth of 5,000; medium: taxa were detected but its abundance is lower than the median of abundance in detected individuals; high: taxa were detected and its abundance is higher than the median of abundance in detected individuals). *P*-values of comparison between two groups were determined by Wilcox tests (two-tailed). * $P < 0.05$, ** $P < 0.01$, *** $P < 0.001$.

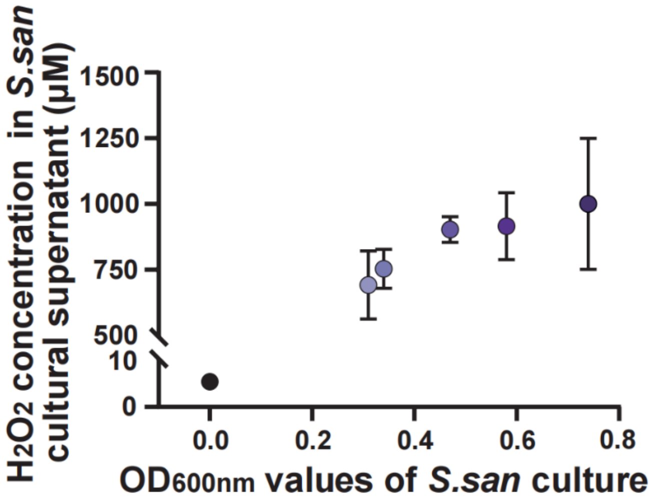


**Supplementary Figure4: $H_2O_2$ concentration of the culture supernatant of *S. sanguinis* after 24 hours of culture.** $OD_{600nm}$: the $OD_{600nm}$ value of the bacteria when harvesting the supernatant. An OD value equal to zero represents the culture medium control.

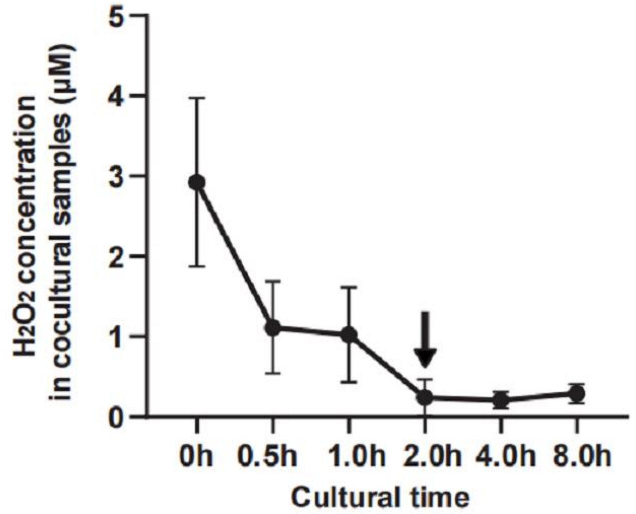


**Supplementary Figure5: the $H_2O_2$ concentration in coculture supernatant with different culture times.** Akata cells were treated with 15μM $H_2O_2$, the supernatant was harvested in 0h, 0.5h, 1.0h, 2.0h, 4.0h and 8.0h after $H_2O_{2was}$ added to measure the levels of $H_2O_2$.

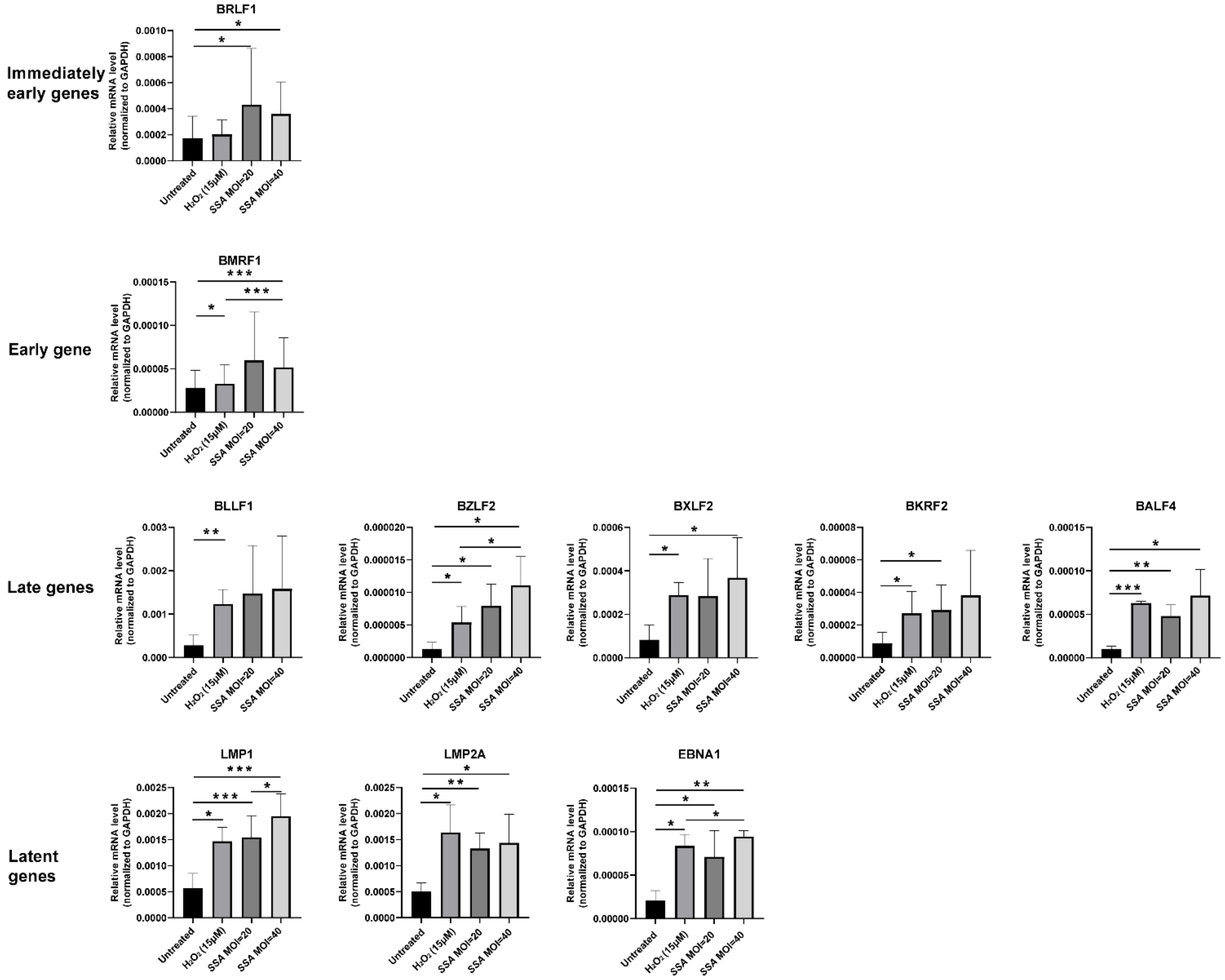


**Supplementary Figure 6: Independent qPCR validation of EBV genes expression of $H_2O_2$ treated and *S.sanguinis* cocultured samples.** Each column represents 3 independent repetitions. *P*-values were determined by unpaired *t* test (two-tailed). **P* < 0.05, ***P* < 0.01, ****P* < 0.001. Data are expressed as mean ± SD.

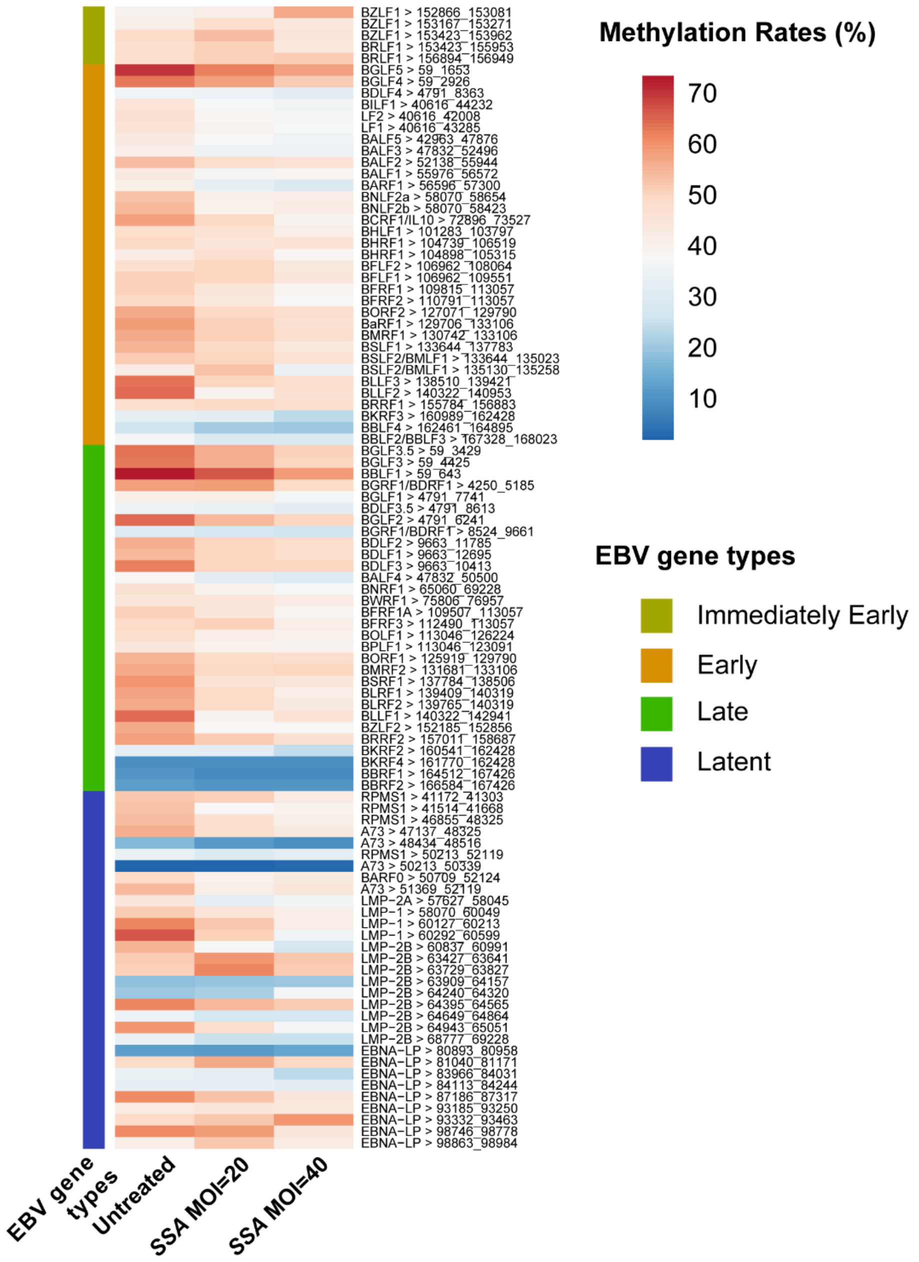


**Supplementary Figure 7: Heatmap of methylation rates of EBV encoded genes in untreated and *S.sanguinis* cocultured Akata cells.** All EBV annotated CpGs from the types of immediately early ($N = 5$), early ($N = 36$), late ($N = 39$) and latent ($N = 30$) genes were shown. Row names represent “gene_name > CpG start position_CpG end position”.

## Appendix 2. Supplementary Tables

**Supplementary Table 1. The demographic characteristics of participants in this study**

| Characteristics | Case(N=150) | Control(N=153) | *P*-value[a] |
|---|---|---|---|
| **Age (years), Mean ± SD** | 48.6 ± 10.1 | 46.9 ± 10.2 | 0.151 |
| **Gender, N (%)** | | | |
| Male | 105(70.0%) | 108(70.6%) | 0.999 |
| Female | 45(30.0%) | 45(29.4%) | |
| **Education, N (%)** | | | 0.204 |
| <High school | 128(85.3%) | 121(79.1%) | |
| ≥High school | 22(14.7%) | 32(20.9%) | |
| **Smoking status, N (%)** | | | 0.049 |
| Current | 67(44.7%) | 74(48.4%) | |
| Former | 27(18.0%) | 13(8.5%) | |
| Never | 56(37.3%) | 66(43.1%) | |
| **Alcohol consumption, N (%)** | | | 0.999 |
| No | 97(64.7%) | 98(64.1%) | |
| Yes | 53(35.3%) | 55(35.9%) | |
| **Teeth loss after age 20, N (%)** | | | 0.443 |
| No | 64 (42.7) | 73 (47.7) | |
| Yes | 86 (57.3) | 80 (52.3) | |
| **Filling teeth, N (%)** | | | 0.372 |
| No | 108 (72.0) | 118 (77.1) | |
| Yes | 42 (28.0) | 35 (22.9) | |
| **Tooth brushing frequency, N (%)** | | | |
| < 2 times/day | 47 (31.3) | 54 (35.3) | 0.542 |
| ≥ 2 times/day | 103 (68.7) | 99 (64.7) | |
| **OD values of EBV VCA-IgA antibody, Mean ± SD [b]** | 4.65 ± 2.59 | 0.55 ± 0.51 | < 0.001 |
| **Sequencing depth (reads/sample), Mean ± SD** | 29848.3 ± 19173.2 | 28678.8 ± 11189.8 | 0.518 |
| **experimental batches, N (%)** | | | 0.549 |
| Batch1 | 49 (32.7%) | 41 (26.8%) | |
| Batch2 | 44 (29.3%) | 47 (30.7%) | |
| Batch3 | 42 (28.0%) | 43 (28.1%) | |
| Batch4 | 15 (10.0%) | 22 (14.4%) | |

[a]*P*-value of continuous variables (age, OD values of EBV VCA-IgA antibody and sequencing depth) were calculated from Welch Two Sample t-test for comparison between those in the case group and those in the control group (two-tailed, t-test function from MASS package, R software); *P*-value from categorical variables (gender, education, smoking status, alcohol consumption, teeth loss after age 20, filling teeth, tooth brushing frequency and experimental batches) were calculated from Pearson's Chi-squared test for comparison between those in case group and those in the control group (chisq.test () from vcd package, R);

[b]OD values of the EBV VCA-IgA antibody of 26 participants were not obtained. OD values of 137 NPC patients and 140 controls were shown.

**Supplementary Table 2. Alpha and beta diversity analysis of oral microbiota and environmental factors**

| Variables | alpha diversity (Shannon index) | | beta diversity (unweighted UniFrac) | | |
|---|---|---|---|---|---|
| | t/F | *p*-value [1] | $R^2$ | F | *p*-value [2] |
| Cigarette smoking status | 4.981 | 0.007 | 0.01 | 1.631 | 0.004 |
| Alcohol consumption | 1.373 | 0.173 | 0.003 | 0.939 | 0.573 |
| Teeth lost after age 20 | 1.938 | 0.054 | 0.005 | 1.514 | 0.034 |
| Teeth brushing frequency | 1.758 | 0.008 | 0.004 | 1.173 | 0.177 |
| Filling teeth | 0.395 | 0.694 | 0.003 | 0.972 | 0.453 |

[1] *P*-values were obtained from *t*-test in variables of sex, education, alcohol consumption, teeth loss after age 20, filling teeth and tooth brushing frequency; from ANOVA of age and cigarette smoking status.

[2] $R^2$, F and *p*-value were obtained from adonis analysis.

**Supplementary Table 3. The relative abundance difference of oral taxa between NPC patients and controls [a]**

| ASVs[b] | Taxonomy | | | Relative abundance (‰) | | Detection rates (%) | | DESeq analysis | |
|---|---|---|---|---|---|---|---|---|---|
| | Phylum | Genus | Species | NPC | Control | NPC | Control | $log_2Fc$ (95%CI) | *P*-value |
| c845 | Proteobacteria | Lautropia | mirabilis | 14.68 | 8.09 | 95.33 | 96.08 | 1.07 (0.85, 1.29) | 6.82E-07 |
| 3216 | Saccharibacteria_(TM7) | Saccharibacteria_(TM7)_[G-5] | HMT_356 | 0.32 | 0.79 | 36.00 | 61.44 | -1.38 (-1.72, -1.04) | 5.31E-05 |
| 37ab | Firmicutes | Oribacterium | asaccharolyticum | 1.25 | 0.48 | 45.33 | 36.60 | 1.73 (1.30, 2.16) | 6.44E-05 |
| 021c | Bacteroidetes | Bacteroidetes_[G-5] | HMT_511 | 0.18 | 0.46 | 24.67 | 45.75 | -1.62 (-2.03, -1.21) | 8.03E-05 |
| e811 | Fusobacteria | Leptotrichia | buccalis | 0.65 | 1.53 | 30.67 | 63.40 | -1.45 (-1.82, -1.08) | 8.10E-05 |
| 5a26 | Actinobacteria | Actinomyces | | 0.89 | 0.42 | 30.00 | 21.57 | 2.06 (1.47, 2.65) | 4.44E-04 |
| ce54 | Bacteroidetes | Capnocytophaga | sputigena | 3.96 | 2.25 | 84.00 | 86.27 | 0.74 (0.52, 0.96) | 9.27E-04 |
| 1f05 | Firmicutes | Peptostreptococcus | stomatis | 2.64 | 1.81 | 89.33 | 96.08 | 0.67 (0.46, 0.88) | 1.50E-03 |
| 1b51 | Firmicutes | Streptococcus | | 5.15 | 3.29 | 74.67 | 64.71 | 0.89 (0.61, 1.17) | 1.56E-03 |
| fcb6 | Saccharibacteria_(TM7) | Saccharibacteria_(TM7)_[G-3] | HMT_351 | 0.71 | 1.22 | 51.33 | 79.08 | -0.88 (-1.16, -0.6) | 1.63E-03 |
| ca9c | Proteobacteria | Haemophilus | | 7.42 | 4.74 | 82.67 | 79.74 | 0.73 (0.48, 0.98) | 3.45E-03 |
| 17ae | Fusobacteria | Leptotrichia | sp._HMT_417 | 3.59 | 5.87 | 63.33 | 86.93 | -0.82 (-1.11, -0.53) | 4.37E-03 |
| 6941 | Gracilibacteria_(GN02) | (GN02)_[G-2] | bacterium_HMT_873 | 0.34 | 0.87 | 16.00 | 25.49 | -1.92 (-2.6, -1.24) | 4.87E-03 |
| b8eb | Proteobacteria | Campylobacter | | 0.48 | 0.94 | 26.67 | 30.72 | -1.58 (-2.15, -1.01) | 5.90E-03 |
| 3941 | Firmicutes | Streptococcus | | 2.51 | 3.53 | 67.33 | 89.54 | -0.55 (-0.75, -0.35) | 6.96E-03 |
| 8f92 | Bacteroidetes | Alloprevotella | rava | 1.96 | 4.28 | 42.00 | 62.09 | -1.14 (-1.57, -0.71) | 8.20E-03 |
| 8194 | Proteobacteria | Haemophilus | parainfluenzae | 1.00 | 0.44 | 32.00 | 15.69 | 1.66 (1.02, 2.30) | 8.90E-03 |
| 394e | Proteobacteria | Haemophilus | parainfluenzae | 74.89 | 63.90 | 99.33 | 100.00 | 0.35 (0.21, 0.49) | 9.19E-03 |
| 2b86 | Fusobacteria | Leptotrichia | sp._HMT_221 | 1.12 | 1.97 | 38.67 | 62.75 | -1.02 (-1.41, -0.63) | 9.39E-03 |
| 0ed3 | Actinobacteria | Actinomyces | | 1.07 | 0.52 | 41.33 | 37.91 | 1.07 (0.66, 1.48) | 9.93E-03 |
| 6cb8 | Firmicutes | Selenomonas | | 0.86 | 1.46 | 50.00 | 79.08 | -0.70 (-0.97, -0.43) | 1.03E-02 |
| 310a | Proteobacteria | Campylobacter | sp._HMT_044 | 0.40 | 0.72 | 34.67 | 49.67 | -1.06 (-1.47, -0.65) | 1.04E-02 |
| 2df3 | Firmicutes | Oribacterium | parvum | 0.71 | 1.10 | 54.67 | 69.93 | -0.73 (-1.02, -0.44) | 1.13E-02 |
| d23d | Firmicutes | Stomatobaculum | sp._HMT_097 | 1.23 | 1.95 | 63.33 | 81.05 | -0.68 (-0.95, -0.41) | 1.17E-02 |
| ad74 | Firmicutes | | | 0.11 | 0.28 | 19.33 | 28.76 | -1.35 (-1.89, -0.81) | 1.31E-02 |
| 8746 | Bacteroidetes | Capnocytophaga | sp._HMT_863 | 2.28 | 3.02 | 60.67 | 81.70 | -0.66 (-0.93, -0.39) | 1.34E-02 |

| | | | | | | | | | |
|---|---|---|---|---|---|---|---|---|---|
| cbef | Bacteroidetes | Prevotella | pallens | 8.25 | 5.28 | 77.33 | 74.51 | 0.75 (0.44, 1.06) | 1.34E-02 |
| 732f | Actinobacteria | Actinomyces | | 1.99 | 3.83 | 26.67 | 64.05 | -1.05 (-1.48, -0.62) | 1.56E-02 |
| fa21 | Bacteroidetes | Bacteroidetes_[G-6] | HMT_516 | 0.11 | 0.21 | 11.33 | 30.07 | -1.34 (-1.9, -0.78) | 1.63E-02 |
| 77a9 | Actinobacteria | Corynebacterium | matruchotii | 1.29 | 1.77 | 68.00 | 85.62 | -0.50 (-0.71, -0.29) | 1.92E-02 |
| c3a4 | Proteobacteria | Campylobacter | | 2.16 | 3.02 | 67.33 | 84.97 | -0.56 (-0.8, -0.32) | 1.93E-02 |
| a375 | Fusobacteria | Leptotrichia | hongkongensis | 0.67 | 0.39 | 54.67 | 43.14 | 0.75 (0.43, 1.07) | 1.96E-02 |
| 1b14 | Gracilibacteria_(GN02) | (GN02)_[G-2] | HMT_873 | 0.79 | 1.00 | 22.67 | 45.10 | -1.21 (-1.73, -0.69) | 2.12E-02 |
| d7f3 | Saccharibacteria_(TM7) | Saccharibacteria_(TM7)_[G-1] | HMT_349 | 0.17 | 0.35 | 18.00 | 38.56 | -1.07 (-1.54, -0.6) | 2.29E-02 |
| 5f73 | Bacteroidetes | Prevotella | intermedia | 0.83 | 1.48 | 21.33 | 29.41 | -1.51 (-2.17, -0.85) | 2.36E-02 |
| 814d | Fusobacteria | Leptotrichia | sp._HMT_215 | 1.57 | 0.55 | 29.33 | 24.84 | 1.41 (0.78, 2.04) | 2.51E-02 |
| f186 | Proteobacteria | Neisseria | | 60.11 | 36.85 | 88.67 | 94.12 | 0.47 (0.25, 0.69) | 2.94E-02 |
| 14be | Firmicutes | | | 0.23 | 0.48 | 24.67 | 50.98 | -0.92 (-1.35, -0.49) | 3.11E-02 |
| f117 | Firmicutes | Selenomonas | | 1.64 | 1.98 | 57.33 | 73.86 | -0.66 (-0.97, -0.35) | 3.32E-02 |
| 8ae8 | Firmicutes | Lachnospiraceae_[G-3] | HMT_100 | 1.13 | 1.50 | 42.67 | 62.09 | -0.79 (-1.17, -0.41) | 3.46E-02 |
| e78e | Proteobacteria | Aggregatibacter | | 10.68 | 13.43 | 82.67 | 98.04 | -0.41 (-0.6, -0.22) | 3.62E-02 |
| e93b | Firmicutes | Parvimonas | micra | 1.97 | 1.23 | 49.33 | 46.41 | 0.86 (0.45, 1.27) | 3.65E-02 |
| 118d | Saccharibacteria_(TM7) | Saccharibacteria_(TM7)_[G-2] | HMT_350 | 0.09 | 0.16 | 14.67 | 34.64 | -0.96 (-1.42, -0.5) | 3.80E-02 |
| 5b92 | Firmicutes | Peptostreptococcaceae_[XI][G-6] | nodatum | 0.08 | 0.19 | 14.67 | 28.76 | -1.04 (-1.54, -0.54) | 3.85E-02 |
| 3916 | Firmicutes | Megasphaera | micronuciformis | 6.73 | 4.69 | 72.00 | 78.43 | 0.61 (0.30, 0.92) | 4.92E-02 |

[a] 45 core ASVs with a detection rate >20% in the case-control population were analyzed; ASVs which $p<0.05$ in DESeq analysis are shown. Adjusting for age, sex, education, smoking status, alcohol drinking, teeth loss after age 20, filling teeth and tooth brushing frequency.

[b] the first 4 letters of the ASV ID are shown.

**Supplementary Table 4. Sequence alignments of *Streptococcus* species with read of *Streptococcus*_ASV.1b51[a]**

| Description | Max Score | E value | Identities (Per.) | Gaps (Per.) | Isolation[b] |
|---|---|---|---|---|---|
| *Streptococcus sanguinis* SK1 = NCTC 7863 | 466 | 3E-131 | 252/252 (100%) | 0/252 (0%) | Infective endocarditis (human) |
| *Streptococcus sanguinis* strain JCM 5708 | 466 | 3E-131 | 252/252 (100%) | 0/252 (0%) | Infective endocarditis (human) |
| *Streptococcus cristatus* AS 1.3089 | 460 | 1E-129 | 251/252 (99.6%) | 0/252 (0%) | Oral cavities (human) |
| *Streptococcus cristatus* ATCC 51100 | 460 | 1E-129 | 251/252 (99.6%) | 0/252 (0%) | Periodontal abscess (human) |
| *Streptococcus cristatus* strain 2-4 | 460 | 1E-129 | 251/252 (99.6%) | 0/252 (0%) | Oral cavities (human) |
| *Streptococcus panodentis* strain TKU50 | 460 | 1E-129 | 251/252 (99.6%) | 0/252 (0%) | Oral cavities (chimpanzees) |
| *Streptococcus sinensis* strain HKU4 | 460 | 1E-129 | 251/252 (99.6%) | 0/252 (0%) | Infective endocarditis (human) |
| *Streptococcus parasanguinis* ATCC 15912 | 457 | 2E-128 | 250/252 (99.2%) | 0/252 (0%) | Throat (human) |
| *Streptococcus gordonii* strain SK3 | 455 | 6E-128 | 250/252 (99.2%) | 0/252 (0%) | Subacute bacterial endocarditis (human) |
| *Streptococcus himalayensis* strain HTS2 | 455 | 6E-128 | 250/252 (99.2%) | 0/252 (0%) | Respiratory tract (Marmota himalayana) |
| *Streptococcus rubneri* strain LMG 27207 | 455 | 6E-128 | 250/252 (99.2%) | 0/252 (0%) | Throat (human) |
| *Streptococcus australis* strain AI-1 | 455 | 6E-128 | 250/252 (99.2%) | 0/252 (0%) | Saliva (human) |

[a]Searching the database of 16S ribosomal RNA sequences (Bacteria and Archaea) using Megablast (Optimize for highly similar sequences)

[b]Source information from NCBI's Nucleotide (title and source), ATCC websites and Bergey's manual of systematic bacteriology.

**Supplementary Table 5. Enriched hallmark pathways and core enrichment genes of RNA-seq [a]**

| Pathways | NES [b] | FDR *q*-value [c] | Leading edge | Core enrichment genes |
|---|---|---|---|---|
| ***SSA* MOI=20 *vs.* Untreated** | | | | |
| HALLMARK_TNFA_SIGNALING_VIA_NFKB | 2.25 | 3.35E-05 | tags=36%, list=12%, signal=40% | CD69,HES1,PPP1R15A,NFKBIA,PNRC1,CXCL10,NR4A1,CD83,BTG1,DUSP5,IFIT2,ACKR3,CEBPB,KLF10,TNFAIP3,GADD45B,FOS,IER2,BCL3,TNFSF9,NFKB2,LITAF,IRF1,SNN,ZBTB10,MYC,PHLDA1,EIF1,SGK1,JUNB,BTG2,DUSP2,SQSTM1,RELB,TRIB1,NFKB1,NFKBIE,IER5,IFNGR2,RIPK2,LAMB3,ZFP36 |
| HALLMARK_MYC_TARGETS_V2 | 2.00 | 1.16E-03 | tags=46%, list=23%, signal=59% | WDR74,NOP2,MYC,BYSL,NIP7,PPAN,DUSP2,PES1,NOLC1,AIMP2,PLK1,SUPV3L1,NOP16,NDUFAF4,RRP9,WDR43,PA2G4,EXOSC5,TBRG4,GNL3,MRTO4,GRWD1,RRP12,PUS1,FARSA,PRMT3 |
| HALLMARK_INTERFERON_GAMMA_RESPONSE | 1.68 | 2.61E-02 | tags=36%, list=22%, signal=46% | CD69,NFKBIA,CXCL10,PIM1,TXNIP,BTG1,RTP4,IFIT2,TNFAIP3,TNFSF10,USP18,IRF1,CD86,IFI44L,SAMD9L,EPSTI1,RNF213,IFIT3,IRF5,CASP4,TRIM21,HIF1A,IRF9,IRF8,NFKB1,PARP12,PSME1,STAT1,RIPK2,UBE2L6,C1S,TRIM25,OASL,ISG15,ICAM1,ZNFX1,IFIT1,SP110,TRAFD1,OAS2,TOR1B,IRF7,IFIH1,DHX58,OGFR,PNP,B2M,PNPT1,PSMB8,MT2A,VAMP5,LY6E |
| HALLMARK_INFLAMMATORY_RESPONSE | 1.61 | 2.85E-02 | tags=25%, list=12%, signal=28% | EBI3,CD69,NFKBIA,CXCL10,RTP4,TNFSF10,TNFSF9,IL10,SLC31A2,IRF1,MYC,KCNA3,ADORA2B,BTG2,CD55,HIF1A,NFKB1,PSEN1,BEST1,IFNGR2,RIPK2 |
| HALLMARK_INTERFERON_ALPHA_RESPONSE | 1.61 | 3.36E-02 | tags=45%, list=21%, signal=57% | CXCL10,TXNIP,RTP4,IFIT2,USP18,IRF1,IFI44L,SAMD9L,EPSTI1,IFIT3,OAS1,TRIM21,SAMD9,IRF9,PARP12,PSME1,PARP9,RIPK2,UBE2L6,C1S,TMEM140,TRIM25,OASL,ISG15,NUB1,SP110,TRAFD1,IRF7,IFIH1,DHX58,NCOA7,OGFR,B2M,PNPT1,PSMB8 |
| HALLMARK_IL6_JAK_STAT3_SIGNALING | 1.55 | 3.90E-02 | tags=20%, list=12%, signal=23% | EBI3,INHBE,CXCL10,PIM1,IRF1,BAK1,IRF9,STAT1,IFNGR2 |
| ***SSA* MOI=20 *vs.* Untreated** | | | | |
| HALLMARK_TNFA_SIGNALING_VIA_NFKB | 2.53 | 0.00E+00 | tags=45%, list=16%, signal=53% | CD69,FOS,DUSP5,PPP1R15A,NFKBIA,CSF1,TNFSF9,PNRC1,HES1,PHLDA1,CXCL10,BTG1,IFIT2,TNFAIP3,ZBTB10,CEBPB,CD83,IRF1,NR4A1,LITAF,DENND5A,ACKR3,ICAM1,KLF10,IER2,GADD45B,NFKB2,SGK1,RELB,MYC,TRIB1,IER5,SQSTM1,VEGFA,ZFP36,KLF6,EIF1,IFIH1,JUNB,DUSP2,NFKBIE,PLEK,SMAD3,CD80,NFAT5,PDE4B,TIPARP,ATP2B1,BCL6,JUN,RIPK2,CCNL1,SNN |
| HALLMARK_INTERFERON_GAMMA_RESPONSE | 2.11 | 9.01E-05 | tags=43%, list=21%, signal=54% | CD69,C1S,NFKBIA,CXCL10,BTG1,IFIT2,IRF5,PIM1,IFI44L,TNFAIP3,USP18,OASL,IRF1,ICAM1,IFIT3,TXNIP,MX2,RTP4,RNF213,IRF9,MT2A,SAMD9L,IFI27,ZNFX1,EPSTI1,IRF7,IFIH1,STAT1,CD86,PML,PARP12,HLA-DQA1,CASP4,TRIM21,IFI35,HIF1A,UBE2L6,ISG15,PSME1,PDE4B,HELZ2,OAS2,IFI44,VAMP5,APOL6,TRIM25,RIPK2,OGFR,LYSMD2,TOR1B,B2M,RBCK1,NOD1,TRIM26,LY6E,IRF8,DHX58,EIF2AK2,CMPK2,PNPT1,SLC25A28,TRAFD1 |

| | | | | |
|---|---|---|---|---|
| HALLMARK_INTERFERON_ALPHA_RESPONSE | 2.13 | 9.41E-05 | tags=55%, list=21%, signal=68% | C1S,CSF1,CXCL10,IFIT2,IFI44L,USP18,OASL,IRF1,IFIT3,TXNIP,SAMD9,RTP4,IRF9,OAS1,SAMD9L,IFI27,EPSTI1,IRF7,IFIH1,PARP12,TRIM21,IFI35,UBE2L6,ISG15,PSME1,HELZ2,IFI44,TRIM25,NCOA7,RIPK2,OGFR,PARP9,B2M,TRIM26,LY6E,ELF1,DHX58,EIF2AK2,CMPK2,PNPT1,SLC25A28,TRAFD1 |
| HALLMARK_IL6_JAK_STAT3_SIGNALING | 1.96 | 7.94E-04 | tags=23%, list=10%, signal=25% | EBI3,INHBE,CSF1,CXCL10,PIM1,IRF1,IRF9,STAT1,BAK1,A2M |
| HALLMARK_HYPOXIA | 1.89 | 1.48E-03 | tags=25%, list=10%, signal=27% | FOS,PPP1R15A,PNRC1,KLHL24,SDC3,CXCR4,DDIT3,BTG1,PIM1,TNFAIP3,DDIT4,LOX,ACKR3,MXI1,GCNT2,RORA,BCL2,CDKN1B,MAP3K1,FOXO3,ZNF292,CITED2,MT2A,ADORA2B,VEGFA,ZFP36,KLF6,NOCT,NR3C1,EXT1 |
| HALLMARK_INFLAMMATORY_RESPONSE | 1.84 | 2.52E-03 | tags=37%, list=21%, signal=46% | EBI3,CD69,NFKBIA,CSF1,TNFSF9,CXCL10,IRF1,STAB1,ICAM1,CCR7,RTP4,MYC,ADORA2B,BEST1,SLC7A1,KLF6,IRF7,KCNA3,HIF1A,PDE4B,ATP2B1,CD55,RIPK2,SLC31A1,PSEN1,ICOSLG,LYN,LY6E,EIF2AK2,IL10,BTG2 |
| HALLMARK_MYC_TARGETS_V2 | 1.77 | 4.51E-03 | tags=47%, list=29%, signal=67% | NOP2,MYC,BYSL,WDR74,PPAN,DUSP2,TFB2M,NIP7,PLK1,WDR43,NOLC1,AIMP2,GRWD1,TBRG4,PES1,SUPV3L1,PUS1,EXOSC5,MRTO4,NOP16,FARSA,PPRC1,LAS1L,GNL3,RRP9,RRP12,PRMT3 |
| HALLMARK_NOTCH_SIGNALING | 1.68 | 1.04E-02 | tags=17%, list=1%, signal=17% | NOTCH2,HES1,MAML2,ARRB1 |
| HALLMARK_TGF_BETA_SIGNALING | 1.56 | 2.87E-02 | tags=38%, list=17%, signal=45% | PPP1R15A,FURIN,SPTBN1,LTBP2,KLF10,BCAR3,FNTA,SKIL,JUNB,BMPR2,SMAD3,ARID4B,TGFBR1,NCOR2,SMAD1 |
| HALLMARK_P53_PATHWAY | 1.51 | 3.83E-02 | tags=22%, list=16%, signal=27% | FOS,PPP1R15A,EPHX1,TNFSF9,DDIT3,BTG1,SLC7A11,TRIB3,DDIT4,CDKN2AIP,TXNIP,PITPNC1,TP63,PVT1,FOXO3,AEN,IER5,ADA,PLK3,BAK1,PPM1D,IRAK1,SP1,TP53,TRIAP1,SLC3A2,JUN,PRKAB1,ZFP36L1,RCHY1,TOB1 |
| **$H_2O_2$ *vs.* Untreated** | | | | |
| HALLMARK_TNFA_SIGNALING_VIA_NFKB | 2.36 | 0 | tags=51%, list=17%, signal=61% | DUSP5,TNFSF9,FOS,CXCL10,PHLDA1,CDKN1A,IRF1,VEGFA,ZFP36,IFIT2,DENND5A,PTGER4,JUN,IL23A,JUNB,FOSB,ZC3H12A,HES1,CEBPB,PTPRE,DDX58,NFKB2,IFIH1,ICOSLG,ICAM1,SMAD3,CLCF1,BTG1,PFKFB3,JAG1,SQSTM1,IER2,PER1,SGK1,ACKR3,NFAT5,PNRC1,B4GALT1,RNF19B,LITAF,TAP1,LAMB3,PDE4B,ZBTB10,IER5,NR4A1,KDM6B,TNIP1,EHD1,CFLAR,RELB,LDLR,PLEK,GADD45B,PPP1R15A,CD69,IFNGR2,CSF1,TRIB1,NINJ1 |
| HALLMARK_INFLAMMATORY_RESPONSE | 1.98 | 3.33E-04 | tags=39%, list=16%, signal=47% | EBI3,IL10,STAB1,CCR7,TNFSF9,KCNA3,CXCL10,CDKN1A,IRF1,BEST1,SLC31A2,PTGER4,IRF7,RTP4,KCNMB2,TAPBP,PTPRE,LCK,ICOSLG,PTAFR,ICAM1,EMP3,TNFSF15,ADORA2B,PDE4B,TNFSF10,LDLR,CD55,CD69,IFNGR2,RHOG,CSF1,LYN |

| | | | | |
|---|---|---|---|---|
| HALLMARK_INTERFERON_ALPHA_RESPONSE | 1.95 | 4.13E-04 | tags=53%, list=24%, signal=70% | CXCL10,IRF1,RSAD2,HELZ2,IFIT2,SAMD9L,IRF7,RTP4,MOV10,IFI35,DHX58,C1S,OASL,IFIH1,STAT2,CMPK2,IFI44L,SAMD9,HLA-C,CD74,OAS1,TAP1,SP110,PARP9,USP18,ISG15,UBA7,ISG20,CSF1,IFI44,TRIM25,ELF1,IFIT3,IRF9,IRF2,ADAR,PARP12,LPAR6,TMEM140,UBE2L6,OGFR |
| HALLMARK_NOTCH_SIGNALING | 1.93 | 4.36E-04 | tags=50%, list=16%, signal=59% | NOTCH2,LFNG,MAML2,NOTCH1,DTX2,HES1,WNT5A,JAG1,ARRB1,PSENEN,DTX4,PPARD |
| HALLMARK_INTERFERON_GAMMA_RESPONSE | 1.92 | 4.43E-04 | tags=33%, list=12%, signal=37% | IRF5,RNF213,MT2A,CXCL10,CDKN1A,IRF1,RSAD2,HELZ2,IFIT2,CASP4,SAMD9L,IRF7,RTP4,IFI35,DHX58,PML,C1S,OASL,TAPBP,DDX58,IFIH1,NOD1,AUTS2,HLA-DQA1,ICAM1,BTG1,STAT2,CD86,APOL6,CMPK2,IFI44L,SOCS1,MX2,ARID5B,RBCK1,CD74,TAP1,SP110,HLA-A,USP18,P2RY14,PDE4B,IFIT1,ISG15,HLA-B,TNFSF10,ISG20 |
| HALLMARK_P53_PATHWAY | 1.89 | 5.48E-04 | tags=30%, list=18%, signal=37% | DDIT4,EPHX1,TNFSF9,FOS,HSPA4L,CDKN1A,NOTCH1,SLC7A11,JUN,TP63,ABAT,PLXNB2,PTPRE,ABHD4,TRIB3,PVT1,FGF13,PITPNC1,DDIT3,ZFP36L1,BTG1,FDXR,SFN,SOCS1,TGFB1,RNF19B,MKNK2,HEXIM1,TAP1,CDK5R1,CTSD,JAG2,IER5,SERTAD3,RAB40C,CDKN2A,PPP1R15A,TOB1,ABCC5,NINJ1,DDB2,CSRNP2 |
| HALLMARK_IL6_JAK_STAT3_SIGNALING | 1.88 | 6.12E-04 | tags=45%, list=17%, signal=55% | EBI3,INHBE,CXCL10,IRF1,JUN,TNFRSF12A,STAT2,TYK2,SOCS1,TNFRSF1A,IL17RA,TGFB1,LTB,CBL,IL12RB1,IFNGR2,PIM1,CSF1,STAT3,HAX1 |
| HALLMARK_HEDGEHOG_SIGNALING | 1.84 | 9.23E-04 | tags=62%, list=21%, signal=78% | VEGFA,PML,THY1,CELSR1,GLI1,TLE3,CDK5R1,PTCH1,HEY1,MYH9,OPHN1,AMOT,NF1 |
| HALLMARK_EPITHELIAL_MESENCHYMAL_TRANSITION | 1.81 | 1.28E-03 | tags=41%, list=15%, signal=48% | NOTCH2,VEGFA,JUN,ABI3BP,THY1,TNFRSF12A,PLOD3,WNT5A,PFN2,ITGB5,ENO2,EMP3,MYLK,SNAI2,PCOLCE,TGFB1,FBLN5,LRP1,FLNA,FSTL3,LOXL2,OXTR,BASP1,GADD45B |
| HALLMARK_MYOGENESIS | 1.70 | 5.49E-03 | tags=49%, list=28%, signal=68% | SORBS3,SORBS1,CKB,GPX3,CDKN1A,NAV2,NOTCH1,MYH3,ITGA7,PLXNB2,FABP3,FOXO4,LPIN1,ITGB5,OCEL1,DAPK2,MYLK,CKMT2,DMD,PYGM,TGFB1,PPFIA4,ERBB3,MYH11,MYH9,GADD45B,DTNA,MYO1C,SYNGR2,TAGLN,PGAM2,SPTAN1,SMTN,ACSL1,MEF2D,ATP6AP1,PRNP,ENO3,HDAC5,TSC2,FGF2,GSN,CHRNG,AK1,GAA |
| HALLMARK_HYPOXIA | 1.69 | 5.73E-03 | tags=35%, list=24%, signal=45% | SDC3,DDIT4,FOS,MT2A,CDKN1A,P4HA2,VEGFA,ZFP36,JUN,CITED2,NDST1,DDIT3,BCL2,RORA,BTG1,ENO2,PFKFB3,CCNG2,ACKR3,KLHL24,ADORA2B,PYGM,PNRC1,EXT1,PPFIA4,ISG20,PPARGC1A,MYH9,PPP1R15A,PIM1,DTNA,SLC6A6,GAPDHS,GPC1,CP,SLC25A1,CDKN1B,SIAH2,PGAM2,MXI1,ALDOA,IDS |
| HALLMARK_KRAS_SIGNALING_DN | 1.66 | 6.89E-03 | tags=45%, list=25%, signal=59% | LFNG,SYNPO,RSAD2,CELSR2,YPEL1,KMT2D,GPR19,MYOT,MAGIX,SGK1,IFI44L,SLC29A3,NTF3,ITGB1BP2,MTHFR,PTPRJ,KCND1,ZC2HC1C,MAST3,SNN,HNF1A,CLSTN3,NR6A1,CD80,PDK2 |

| | | | | |
|---|---|---|---|---|
| HALLMARK_IL2_STAT5_SIGNALING | 1.65 | 6.98E-03 | tags=33%, list=19%, signal=41% | IL10,SYT11,CAPN3,FURIN,CXCL10,PHLDA1,TNFRSF8,DENND5A,NFKBIZ,BCL2,RORA,AGER,CD86,IKZF4,PLEC,GPX4,SOCS1,GALM,LTB,HUWE1,TNFSF10,PTCH1,CTSZ,FLT3LG,SLC1A5,AMACR,GADD45B,PIM1,CSF1,MAPKAPK2,POU2F1,MYO1C,TLR7,SLC29A2,SYNGR2,IL10RA |
| HALLMARK_WNT_BETA_CATENIN_SIGNALING | 1.65 | 7.37E-03 | tags=42%, list=16%, signal=50% | NOTCH1,NCOR2,FRAT1,TCF7,WNT5B,JAG1,JAG2,MAML1,PTCH1,HEY1,NOTCH4,PPARD,NUMB |
| HALLMARK_ESTROGEN_RESPONSE_EARLY | 1.66 | 7.41E-03 | tags=41%, list=23%, signal=54% | RPS6KA2,ANXA9,ELF3,FOS,PAPSS2,NAV2,CELSR2,NCOR2,ABAT,HES1,CELSR1,TJP3,WFS1,AFF1,ALDH3B1,MAST4,BCL2,SYNGR1,SLC22A5,SLC37A1,FASN,UNC119,FAM102A,SFN,CBFA2T3,B4GALT1,SLC7A5,BLVRB,KDM4B,MED13L,TOB1,NADSYN1,RARA,FRK,ITPK1,ELF1,PODXL,DHCR7,FDFT1,FARP1,GLA,SIAH2,TIAM1,PMAIP1,TUBB2B |
| HALLMARK_APOPTOSIS | 1.62 | 8.88E-03 | tags=31%, list=18%, signal=38% | GPX3,BMF,CDKN1A,IRF1,NEDD9,CASP4,JUN,CTH,TNFRSF12A,BCL2L11,DDIT3,ENO2,FDXR,SQSTM1,GPX4,LGALS3,SATB1,TAP1,PDCD4,TNFSF10,ISG20,CDC25B,CFLAR,ERBB3,RNASEL,GADD45B,CD69,BCL2L2,RARA,DAP,PPP2R5B,CREBBP |
| HALLMARK_APICAL_JUNCTION | 1.62 | 9.21E-03 | tags=29%, list=21%, signal=36% | SDC3,SORBS3,NECTIN1,NECTIN2,ZYX,WNK4,NRXN2,JUP,THY1,ICAM1,NEXN,INSIG1,CD86,ITGA10,B4GALT1,LAMB3,PECAM1,ATP1A3,MMP9,IRS1,MYH9,MAPK11,CNN2,IKBKG,ICAM5,THBS3,STX4,INPPL1,VCL,NF1,TSPAN4 |
| HALLMARK_TGF_BETA_SIGNALING | 1.60 | 1.05E-02 | tags=35%, list=19%, signal=43% | SPTBN1,FURIN,NCOR2,JUNB,SMAD3,ID1,TGFB1,SMAD6,PPP1R15A,IFNGR2,SKI,BCAR3,TGFBR1,ENG |
| HALLMARK_COMPLEMENT | 1.56 | 1.43E-02 | tags=42%, list=26%, signal=56% | DUSP5,APOBEC3F,CASP10,DGKG,IRF1,FYN,CASP4,IRF7,SPOCK2,C1S,CTSB,CEBPB,CBLB,SH2B3,APOBEC3G,LCK,PCLO,BRPF3,LGALS3,HSPA1A,CDK5R1,LRP1,CTSD,EHD1,CD55,PLEK,NOTCH4,PIM1,RHOG,LYN,ERAP2,STX4,CP,IRF2,S100A13,GNB2,MSRB1,WAS,USP14,RABIF,LGMN,ZEB1,PIK3CG,DOCK9,PSEN1 |
| HALLMARK_CHOLESTEROL_HOMEOSTASIS | 1.49 | 2.82E-02 | tags=54%, list=31%, signal=78% | TRIB3,TNFRSF12A,TP53INP1,JAG1,FASN,LGALS3,PNRC1,ATF5,SREBF2,LDLR,STX5,DHCR7,FDFT1,FADS2,SQLE,LGMN,MVK,HMGCS1,HMGCR,NSDHL,HSD17B7,MVD,ATXN2,CBS,LSS,PCYT2,SCD |
| HALLMARK_ALLOGRAFT_REJECTION | 1.48 | 2.97E-02 | tags=38%, list=25%, signal=49% | |
| HALLMARK_MYC_TARGETS_V1 | -2.81 | 0 | tags=67%, list=30%, signal=95% | PCNA,MRPS18B,PSMA7,PABPC4,UBE2L3,SF3B3,PRPF31,POLD2,TUFM,HNRNPC,RSL1D1,PSMD7,HNRNPA3,DEK,EIF4H,EIF4G2,AIMP2,KPNB1,PPIA,NDUFAB1,MRPL9,NHP2,HPRT1,NOLC1,CDK4,NOP16,RPS3,XRCC6,USP1,PGK1,SNRPD2,CLNS1A,VBP1,HNRNPU,PSMD14,XPO1,RPS10,CTPS1,DHX15,RANBP1,PRPS2,TOMM70,YWHAE,CYC1,HDDC2,RUVBL2,PSMA2,EEF1B2,RPS6,COPS5,RAD23B,PA2G4,CCNA2,SNRPD3,EIF3D,RPL22,GSPT1,NME1,HDAC2,MCM4,CCT4,RPL6,UBA2,EIF1AX,SNRPB2,VDAC3,CCT3,NCBP1,SRPK1,RPL34,SRSF2,CD |

| | | | | |
|---|---|---|---|---|
| | | | | C20,CBX3,POLE3,SRSF1,G3BP1,SET,TARDBP,PTGES3,PRDX3,MAD2L1,CNBP,ILF2,MCM6,NCBP2,C1QBP,EIF3B,LSM7,GNL3,KPNA2,EIF3J,PSMC6,HNRNPA1,EIF4A1,RAN,ETF1,APEX1,NOP56,PRDX4,ABCE1,SSB,AP3S1,CCT7,CCT5,GLO1,HSP90AB1,TRA2B,SNRPG,HSPD1,DDX21,VDAC1,TCP1,SYNCRIP,CANX,CCT2,SSBP1,SNRPA1,SERBP1,NPM1,EIF4E,EIF2S1,RRP9,LDHA,HSPE1,SRSF3,SNRPD1,DDX18,U2AF1,PWP1,ODC1,EXOSC7,SRSF7 |
| HALLMARK_MYC_TARGETS_V2 | -2.29 | 6.37E-06 | tags=49%, list=21%, signal=62% | IMP4,DUSP2,PA2G4,RCL1,PLK4,PRMT3,MCM4,NOC4L,MPHOSPH10,CBX3,DCTPP1,NIP7,GNL3,WDR43,UNG,NOP56,PES1,IPO4,RABEPK,NDUFAF4,HSPD1,TFB2M,UTP20,MRTO4,NPM1,RRP9,HSPE1,DDX18 |
| HALLMARK_E2F_TARGETS | -2.03 | 3.41E-04 | tags=61%, list=35%, signal=93% | MCM3,DONSON,SMC3,TOP2A,ORC2,KIF2C,SPAG5,HELLS,MSH2,AURKB,NAA38,DSCC1,HNRNPD,RAD51AP1,RRM2,IPO7,CDC25A,DCK,RBBP7,GINS3,ATAD2,TMPO,ORC6,PCNA,TP53,RAD50,POLD2,RAD51C,HMGB3,MXD3,DEK,BUB1B,RFC2,EXOSC8,NOLC1,DDX39A,NUDT21,LMNB1,CDK4,CDCA3,MLH1,XRCC6,USP1,CNOT9,UBR7,LUC7L3,CKS1B,DLGAP5,STAG1,CCNB2,XPO1,ANP32E,CTPS1,HMGB2,SMC6,UBE2S,PMS2,RANBP1,BIRC5,RAD1,PPP1R8,EZH2,CCNE1,BARD1,CDK1,PRIM2,PA2G4,PLK4,NBN,POP7,GSPT1,NME1,LBR,ASF1A,MCM4,NUP205,PRPS1,ZW10,MMS22L,SRSF2,CDC20,CHEK1,DCTPP1,SRSF1,GINS4,TRIP13,NASP,RPA2,MAD2L1,CKS2,CSE1L,ING3,MCM6,SLBP,EED,PNN,RFC3,KPNA2,UBE2T,PSMC3IP,PAICS,UNG,DEPDC1,PHF5A,RAN,NOP56,PRDX4,AURKA,AK2,TRA2B,TIPIN,SYNCRIP,GINS1,NUP107,TFRC,HMMR,LYAR,EIF2S1,CDKN3,PTTG1 |
| HALLMARK_OXIDATIVE_PHOSPHORYLATION | -1.68 | 1.05E-02 | tags=51%, list=34%, signal=76% | MRPS30,COX7A2,SUCLG1,ISCU,CS,RHOT1,DLD,ETFA,HSD17B10,AFG3L2,COX17,NDUFB3,SLC25A5,NDUFC1,BDH2,NDUFB4,SUCLA2,NDUFB7,UQCRB,NDUFA2,MDH2,HSPA9,NDUFAB1,ATP6V1E1,DLAT,NDUFA8,NDUFA4,DLST,FH,ATP6V1C1,MRPS22,ABCB7,TIMM50,MRPL11,NDUFA9,TIMM10,TIMM9,ISCA1,RHOT2,IDH3A,TOMM70,MTX2,CYC1,SLC25A11,MTRR,POLR2F,SURF1,AIFM1,ATP6V0E1,NDUFS1,PMPCA,MRPL15,TIMM8B,UQCR10,COX10,OAT,OPA1,SDHB,NDUFB5,LRPPRC,TIMM13,SDHD,MRPS12,NDUFV2,COX11,MPC1,ATP1B1,VDAC3,ACADSB,FDX1,GRPEL1,UQCRC2,MDH1,ACADM,NDUFS4,PRDX3,HCCS,LDHB,FXN,ATP6V1D,MTRF1,TOMM22,PDHB,TIMM17A,VDAC2,MRPL35,CYCS,NDUFA5,VDAC1,CYB5A,LDHA |
| **IgG *vs.* Untreated** | | | | |
| HALLMARK_TNFA_SIGNALING_VIA_NFKB | 2.34 | 0 | tags=46%, list=11%, signal=51% | EGR1,JUN,FOS,NR4A3,FOSB,DUSP5,IER5,EGR2,TNF,PTGER4,PPP1R15A,BCL2A1,CD69,JUNB,ZFP36,PHLDA1,IER2,SGK1,HES1,IER3,BTG1,GADD45B,SMAD3,ID2,MAP3K8,ZC3H12A,KDM6B,SLC2A3,DDX58,FOSL1,CLCF1,SAT1,SQSTM1,IRF1,GADD45A,IL23A,GPR183,DUSP2,ATP2B1,JAG1,ICOSLG,NFAT5,MXD1,MAP2K3,PER1,PNRC1,TNFAIP3,KLF6,SLC16A6,TNIP2,CEBPB,NFIL3,TRIB1 |
| HALLMARK_EPITHELIAL_MESENCHYMAL_TRANSITION | 1.79 | 5.97E-03 | tags=32%, list=10%, signal=35% | NT5E,JUN,CXCL8,LAMA3,TNFRSF12A,FLNA,ABI3BP,COL11A1,GADD45B,ID2,LOX,SAT1,EMP3,GADD45A,TPM4,LRP1,CADM1,OXTR,ACTA2,TNFAIP3 |

| | | | | |
|---|---|---|---|---|
| HALLMARK_MYC_TARGETS_V1 | -2.05 | 5.90E-04 | tags=64%, list=37%, signal=101% | AIMP2,SSBP1,STARD7,NOLC1,PSMB2,PSMB3,HSPD1,MCM4,RSL1D1,PSMD1,POLE3,XRCC6,EIF4E,HNRNPC,COX5A,PSMD8,NDUFAB1,PSMA6,CDC45,PRPF31,DEK,SMARCC1,TRA2B,ORC2,NPM1,PPM1G,ERH,VBP1,YWHAE,EEF1B2,EXOSC7,NOP56,RFC4,PWP1,RPL34,XPO1,TOMM70,CCNA2,CUL1,CDC20,MCM2,CBX3,PCNA,RPL22,DDX21,PPIA,PSMA2,APEX1,EIF2S1,EIF3B,MRPL9,SRSF3,RRM1,HSP90AB1,ABCE1,LSM7,LDHA,PSMD3,HDGF,TCP1,MCM7,U2AF1,SNRPD1,USP1,UBE2L3,ILF2,RPL6,KPNB1,GNL3,CCT4,TFDP1,RRP9,ACP1,SET,XPOT,RPS6,CCT3,SRPK1,TXNL4A,CCT5,RAN,G3BP1,TRIM28,PRDX3,RUVBL2,C1QBP,SERBP1,DHX15,SF3B3,EIF3D,EIF4H,CNBP,CCT7,EIF2S2,PRDX4,CLNS1A,MRPL23,GOT2,UBA2,RPLP0,RPL18,NME1,RPS5,RPL14,RNPS1,MYC,PA2G4,PHB,NHP2,LSM2,RPS3,SNRPA,RPS2,POLD2,GLO1,RPS10,MAD2L1,PGK1,FBL,RACK1,HNRNPA1,IMPDH2,MRPS18B,VDAC1,CYC1,TUFM |
| HALLMARK_OXIDATIVE_PHOSPHORYLATION | -1.72 | 1.16E-02 | tags=59%, list=36%, signal=91% | SLC25A20,GRPEL1,SLC25A5,TIMM8B,NDUFC2,COX5A,NDUFAB1,NDUFS4,COX7B,SLC25A4,COX6B1,OGDH,RETSAT,NDUFB4,COX4I1,NDUFA9,NDUFS6,UQCR11,SLC25A12,FXN,NDUFA4,SDHA,IDH1,TOMM70,LRPPRC,HSPA9,FH,UQCRH,ECH1,IMMT,ACO2,UQCRFS1,TIMM17A,COX11,IDH3B,SUPV3L1,HADHA,LDHA,ETFDH,TIMM50,HSD17B10,NDUFC1,SUCLG1,NDUFA5,NDUFB7,SDHD,AFG3L2,ECI1,MTRF1,ALDH6A1,MRPS12,COX8A,SDHB,MDH2,PDHA1,MRPS11,MRPL15,NDUFA3,TIMM9,NDUFB5,MRPL11,NDUFS2,VDAC2,PRDX3,TOMM22,ATP6V0B,COX7A2L,ISCA1,ATP6V1F,NDUFA2,DLAT,RHOT1,COX6A1,MRPS15,NDUFB8,LDHB,NDUFV2,TCIRG1,NDUFS1,GOT2,HTRA2,GPI,ACADM,COX5B,OXA1L,UQCRC1,NDUFS8,PDHB,COX15,NDUFV1,SLC25A11,COX7A2,MRPL34,CS,MTX2,UQCR10,ABCB7,TIMM13,NDUFS7,CYB5A,BDH2,NDUFA7,VDAC1,CYC1,BCKDHA |
| HALLMARK_E2F_TARGETS | -1.74 | 1.43E-02 | tags=55%, list=36%, signal=84% | MCM4,MXD3,XRCC6,RFC1,ORC6,CDCA3,DLGAP5,CDK1,NBN,DEK,TRA2B,DCLRE1B,RAD51C,CKS2,ORC2,PMS2,TIPIN,CNOT9,PTTG1,ATAD2,MLH1,CHEK1,NOP56,CCNB2,SMC6,MELK,ASF1A,XPO1,CDC20,SLBP,AK2,ING3,MCM2,RAD1,PCNA,EIF2S1,CSE1L,TCF19,AURKA,DNMT1,UBE2S,ESPL1,TBRG4,RAD21,RAD51AP1,KIF22,DDX39A,SUV39H1,ILF3,CDC25A,MCM7,CDKN1A,KIF2C,GINS3,TUBB,USP1,POLD3,IPO7,SSRP1,CBX5,DSCC1,MYBL2,RPA2,EXOSC8,CKS1B,POLD1,AURKB,RAN,RFC3,HMMR,DEPDC1,BIRC5,LIG1,LUC7L3,SNRPB,DCK,MCM3,POP7,NAA38,PRDX4,RFC2,BUB1B,NME1,PAICS,RBBP7,LBR,RNASEH2A,HMGB2,EZH2,ANP32E,STMN1,MYC,PA2G4,SHMT1,POLD2,PRPS1,GINS1,DCTPP1,MAD2L1,CDC25B,SPC24,UBE2T,TUBG1,PAN2,MTHFD2,HMGA1,CHEK2 |

[a] Gene Set Enrichment Analysis (GSEA) was used to identify genesets associated with *S.sanguinis* and $H_2O_2$ treatment with 1,000,000 permutations.

[b] NES (Normalized Enrichment Score) is a statistic of GSEA that score for the gene set after it has been normalized across analyzed gene sets.

[c] A *p*-value of zero, which indicating an actual p value of less than 1/number-of-permutations, that is 1e-6.

**Supplementary Table 6. Differential expression genes of EBV in in coculture samples**[a]

| Genes | Gene type[b] | $H_2O_2$ *vs.* Untreated | | *SSA*-MOI20 *vs.* Untreated | | *SSA*-MOI40 *vs.* Untreated | |
|---|---|---|---|---|---|---|---|
| | | $log_2FC$ | *P*-value | $log_2FC$ | *P*-value | $log_2FC$ | *P*-value |
| BRLF1 | IE | - | - | 0.99 | 1.55E-05 | 1.49 | 1.11E-10 |
| BZLF1 | IE | - | - | - | - | 1.30 | 1.08E-06 |
| BALF1 | E | 1.40 | 1.60E-04 | - | - | 1.48 | 3.69E-05 |
| BALF2 | E | - | - | 0.98 | 7.50E-06 | 1.14 | 2.24E-07 |
| BALF3 | E | - | - | 0.91 | 1.78E-05 | 0.90 | 2.66E-05 |
| BALF5 | E | - | - | 0.72 | 5.57E-04 | 0.92 | 1.35E-05 |
| BARF1 | E | 1.38 | 2.91E-05 | 1.09 | 1.66E-07 | 1.20 | 1.31E-08 |
| BBLF2/BBLF3 | E | - | - | 1.11 | 6.75E-07 | 1.35 | 2.02E-09 |
| BBLF4 | E | - | - | - | - | 1.35 | 7.84E-08 |
| BcRF1 | E | 0.99 | 3.13E-04 | 1.20 | 3.08E-06 | 1.72 | 1.63E-11 |
| BDLF4 | E | - | - | 0.91 | 1.61E-05 | 0.92 | 1.80E-05 |
| BFLF1 | E | 0.84 | 6.76E-04 | 1.15 | 6.31E-07 | 1.50 | 1.06E-10 |
| BFLF2 | E | - | - | 1.26 | 4.85E-07 | 1.55 | 8.98E-10 |
| BFRF1 | E | - | - | 1.07 | 3.04E-07 | 1.13 | 9.31E-08 |
| BFRF2 | E | 0.79 | 4.84E-04 | 1.18 | 2.63E-08 | 1.21 | 1.62E-08 |
| BGLF4 | E | 1.04 | 1.01E-05 | 1.08 | 8.51E-07 | 1.25 | 1.76E-08 |
| BGLF5 | E | 1.11 | 8.97E-06 | 1.29 | 3.28E-08 | 1.40 | 3.24E-09 |
| BHLF1 | E | 0.98 | 8.90E-06 | 1.07 | 1.83E-07 | 1.15 | 3.50E-08 |
| BHRF1 | E | - | - | 0.92 | 1.27E-05 | 1.20 | 2.21E-08 |
| BILF1 | E | 0.8 | 4.66E-04 | 0.85 | 5.20E-05 | 1.04 | 1.18E-06 |
| BKRF3 | E | 0.8 | 6.66E-04 | 1.10 | 5.31E-07 | 1.36 | 9.13E-10 |
| BLLF2 | E | - | - | 1.41 | 8.09E-06 | 1.82 | 4.61E-09 |
| BLLF3 | E | - | - | 1.24 | 7.86E-06 | 1.46 | 1.48E-07 |
| BMRF1 | E | - | - | 1.09 | 1.93E-07 | 1.17 | 3.14E-08 |
| BNLF2a | E | - | - | 1.24 | 4.32E-06 | 1.29 | 2.61E-06 |
| BNLF2b | E | - | - | 1.22 | 2.14E-05 | 1.23 | 2.58E-05 |
| BORF2 | E | 0.91 | 6.80E-05 | 1.11 | 1.75E-07 | 1.27 | 3.51E-09 |
| BRRF1 | E | 1.14 | 2.20E-05 | 1.03 | 4.87E-05 | 1.71 | 1.20E-11 |
| BSLF1 | E | 1.13 | 1.98E-06 | 1.27 | 1.10E-08 | 1.61 | 8.15E-13 |
| BSLF2/BMLF1 | E | - | - | 1.14 | 6.16E-07 | 1.52 | 4.52E-11 |
| BXLF1 | E | 0.97 | 2.94E-05 | 0.84 | 1.00E-04 | 1.08 | 6.96E-07 |
| LF1 | E | 0.84 | 2.82E-04 | - | - | 0.93 | 2.21E-05 |
| LF2 | E | 0.83 | 5.60E-04 | - | - | 0.81 | 3.75E-04 |
| LF3 | E | 0.92 | 4.53E-05 | 0.88 | 2.39E-05 | 1.04 | 9.26E-07 |
| BALF4 | L | - | - | 0.96 | 7.02E-06 | 0.89 | 4.15E-05 |
| BBLF1 | L | 1.25 | 2.88E-05 | 1.35 | 1.59E-06 | 1.36 | 1.70E-06 |
| BBRF1 | L | - | - | - | - | 1.15 | 5.37E-07 |

| | | | | | | | |
|---|---|---|---|---|---|---|---|
| BBRF2 | L | - | - | - | - | 0.93 | 3.67E-04 |
| BBRF3 | L | - | - | 0.77 | 7.76E-04 | 1.03 | 7.25E-06 |
| BcLF1 | L | 0.81 | 5.59E-04 | 1.04 | 2.04E-06 | 1.29 | 6.19E-09 |
| BCRF1/IL10 | L | 1.90 | 2.71E-07 | 1.84 | 2.29E-07 | - | - |
| BDLF1 | L | - | - | 1.25 | 1.85E-09 | 1.22 | 7.98E-09 |
| BDLF2 | L | - | - | 1.18 | 1.75E-08 | 1.16 | 3.92E-08 |
| BDLF3 | L | - | - | 1.19 | 4.24E-08 | 1.15 | 1.56E-07 |
| BDLF3.5 | L | - | - | 0.92 | 1.46E-05 | 0.92 | 1.77E-05 |
| BdRF1 | L | 0.91 | 2.48E-04 | 0.80 | 5.84E-04 | 0.96 | 4.49E-05 |
| BFRF1A | L | 0.77 | 6.30E-04 | 1.09 | 1.96E-07 | 1.15 | 6.04E-08 |
| BFRF3 | L | - | - | 1.18 | 7.15E-08 | 1.16 | 1.83E-07 |
| BGLF1 | L | - | - | 0.95 | 8.25E-06 | 0.95 | 1.01E-05 |
| BGLF2 | L | - | - | 0.97 | 6.74E-06 | 0.95 | 1.52E-05 |
| BGLF3 | L | 0.95 | 4.24E-05 | 1.05 | 1.15E-06 | 1.26 | 7.87E-09 |
| BGLF3.5 | L | 0.95 | 4.27E-05 | 1.03 | 2.15E-06 | 1.21 | 3.50E-08 |
| BGRF1/BDRF1 | L | - | - | 0.90 | 8.02E-05 | 0.9 | 1.09E-04 |
| BILF2 | L | 1.12 | 9.03E-06 | 1.38 | 5.31E-09 | 1.51 | 2.50E-10 |
| BKRF2 | L | - | - | 1.00 | 3.31E-06 | 1.23 | 1.71E-08 |
| BKRF4 | L | - | - | 1.10 | 2.11E-06 | 1.27 | 7.13E-08 |
| BLLF1 | L | - | - | 1.06 | 3.41E-06 | 1.25 | 4.90E-08 |
| BLRF1 | L | 0.92 | 2.99E-04 | 1.40 | 2.87E-09 | 1.54 | 1.31E-10 |
| BLRF2 | L | - | - | 1.42 | 7.01E-09 | 1.54 | 4.37E-10 |
| BMRF2 | L | - | - | 1.15 | 7.22E-08 | 1.27 | 4.94E-09 |
| BNRF1 | L | - | - | 1.03 | 1.14E-06 | 1.30 | 1.35E-09 |
| BOLF1 | L | 0.97 | 2.55E-05 | 0.97 | 5.31E-06 | 1.43 | 4.49E-11 |
| BORF1 | L | 0.81 | 3.21E-04 | 1.00 | 2.01E-06 | 1.21 | 1.47E-08 |
| BPLF1 | L | 1.02 | 1.18E-05 | 1.03 | 2.14E-06 | 1.46 | 2.75E-11 |
| BRRF2 | L | - | - | 0.83 | 6.50E-04 | 1.03 | 2.88E-05 |
| BSRF1 | L | - | - | 1.05 | 1.95E-04 | 1.29 | 5.21E-06 |
| BTRF1 | L | - | - | - | - | 1.51 | 4.91E-06 |
| BVLF1 | L | 0.96 | 1.78E-04 | - | - | 0.88 | 2.82E-04 |
| BVRF1 | L | 1.11 | 4.66E-06 | 0.76 | 8.04E-04 | 1.15 | 5.15E-07 |
| BVRF2 | L | 1.01 | 2.47E-05 | 0.89 | 6.35E-05 | 1.06 | 2.89E-06 |
| BWRF1 | L | - | - | - | - | - | - |
| BXLF2 | L | 1.02 | 9.34E-05 | 1.38 | 1.23E-08 | 1.67 | 9.27E-12 |
| BXRF1 | L | 1.19 | 4.46E-07 | 0.8 | 3.09E-04 | 1.18 | 1.25E-07 |
| BZLF2 | L | 1.29 | 2.66E-04 | 1.63 | 5.72E-07 | 1.43 | 2.29E-05 |
| A73 | LT | - | - | - | - | 0.83 | 1.54E-04 |
| BARF0 | LT | 0.95 | 1.49E-04 | - | - | - | - |
| EBNA-LP | LT | - | - | - | - | 1.33 | 5.93E-05 |
| LMP-1 | LT | 0.91 | 7.61E-04 | 1.21 | 1.33E-06 | 1.54 | 1.04E-09 |

| | | | | | | | |
|---|---|---|---|---|---|---|---|
| LMP-2A | LT | 1.19 | 1.89E-06 | 1.17 | 6.16E-07 | 1.44 | 1.15E-09 |
| LMP-2B | LT | 1.12 | 9.02E-06 | 1.17 | 7.20E-07 | 1.53 | 1.27E-10 |
| RPMS1 | LT | 0.83 | 2.56E-04 | - | - | 0.91 | 2.00E-05 |
| Cp_Promoter | Cp | - | - | - | - | - | - |
| Cp-EBNA1 | Cp | - | - | 1.13 | 4.78E-06 | 1.45 | 4.51E-09 |
| Cp-EBNA2 | Cp | 1.10 | 8.18E-05 | 0.92 | 4.90E-04 | 1.49 | 1.31E-08 |
| Cp-EBNA3A | Cp | 1.07 | 1.53E-05 | 1.02 | 9.68E-06 | 1.45 | 3.73E-10 |
| Cp-EBNA3B | Cp | 0.84 | 3.30E-04 | 1.16 | 1.13E-07 | 1.38 | 4.48E-10 |
| Cp-EBNA3C | Cp | - | - | 1.26 | 4.52E-08 | 1.41 | 1.34E-09 |
| Qp-EBNA1 | Qp | 1.35 | 2.09E-06 | 1.24 | 4.92E-06 | 1.73 | 1.12E-10 |
| ebv-miR-BART10 | miR | - | - | - | - | - | - |
| ebv-miR-BART11-3p | miR | - | - | - | - | - | - |
| ebv-miR-BART11-5p | miR | - | - | - | - | - | - |
| ebv-miR-BART12 | miR | - | - | - | - | - | - |
| ebv-miR-BART13 | miR | - | - | - | - | - | - |
| ebv-miR-BART1-3p | miR | - | - | - | - | - | - |
| ebv-miR-BART14 | miR | - | - | - | - | - | - |
| ebv-miR-BART15 | miR | - | - | - | - | - | - |
| ebv-miR-BART1-5p | miR | - | - | - | - | - | - |
| ebv-miR-BART16 | miR | - | - | - | - | - | - |
| ebv-miR-BART17-3p | miR | - | - | - | - | - | - |
| ebv-miR-BART17-5p | miR | - | - | - | - | - | - |
| ebv-miR-BART18-3p | miR | - | - | - | - | - | - |
| ebv-miR-BART19-3p | miR | - | - | - | - | - | - |
| ebv-miR-BART19-5p | miR | - | - | - | - | - | - |
| ebv-miR-BART20-3p | miR | - | - | - | - | - | - |
| ebv-miR-BART20-5p | miR | - | - | - | - | - | - |
| ebv-miR-BART21-3p | miR | - | - | - | - | - | - |
| ebv-miR-BART21-5p | miR | - | - | - | - | - | - |
| ebv-miR-BART22 | miR | - | - | - | - | - | - |
| ebv-miR-BART2-3p | miR | 1.23 | 4.72E-04 | 1.60 | 7.41E-07 | 1.34 | 6.94E-05 |
| ebv-miR-BART2-5p | miR | - | - | 1.42 | 8.08E-06 | 1.20 | 2.93E-04 |
| ebv-miR-BART3 | miR | - | - | - | - | - | - |
| ebv-miR-BART4 | miR | - | - | - | - | - | - |
| ebv-miR-BART5 | miR | - | - | - | - | - | - |
| ebv-miR-BART6-3p | miR | - | - | - | - | - | - |
| ebv-miR-BART6-5p | miR | - | - | - | - | - | - |
| ebv-miR-BART7 | miR | - | - | - | - | - | - |
| ebv-miR-BART8 | miR | - | - | - | - | - | - |
| ebv-miR-BART9 | miR | - | - | - | - | - | - |
| ebv-miR-BHRF1-1 | miR | - | - | - | - | - | - |

| | | | | | | | |
|---|---|---|---|---|---|---|---|
| ebv-miR-BHRF1-2 | miR | - | - | - | - | - | - |
| ebv-miR-BHRF1-3 | miR | - | - | - | - | - | - |
| EBER1 | others | - | - | 0.79 | 1.10E-04 | - | - |
| EBER2 | others | - | - | - | - | - | - |
| OriLyt | others | 0.90 | 5.80E-05 | 0.86 | 3.48E-05 | 1.02 | 1.33E-06 |
| OriP | others | 1.47 | 2.92E-06 | 1.40 | 2.73E-06 | 1.70 | 1.17E-08 |
| IR1_W_repeats | others | - | - | - | - | - | - |
| IR2/NotI_repeats | others | 1.02 | 6.05E-06 | 1.36 | 8.35E-11 | 1.20 | 1.46E-08 |
| Repeat_family_type_A | others | - | - | 1.48 | 2.46E-05 | - | - |
| Repeat_family_type_B | others | - | - | - | - | - | - |
| Repeat_family_type_C | others | - | - | 1.61 | 4.45E-06 | - | - |
| Repeat_family_type_D | others | - | - | - | - | - | - |
| Repeat_family-IR3 | others | - | - | - | - | - | - |
| Repeat_IR4_PstI | others | 0.93 | 3.63E-05 | 0.89 | 2.01E-05 | 1.05 | 6.48E-07 |
| Repeat_region | others | 0.98 | 5.67E-04 | - | - | 1.08 | 6.43E-05 |
| Repeat_unit_range-36294..36302 | others | - | - | - | - | - | - |
| Repeat_unit_range-37138..37143 | others | - | - | - | - | - | - |
| Repeat_unit_range-57298..57348 | others | 1.55 | 6.59E-05 | - | - | 1.96 | 6.11E-08 |
| Repeat_unit_range-58001..58015 | others | - | - | - | - | - | - |
| Repeat_unit_range67452..167484 | others | - | - | - | - | - | - |
| Repeat_unit_range-69852..69922 | others | - | - | - | - | - | - |
| Repeat_unit_range-71173..71181 | others | - | - | 1.25 | 1.43E-04 | 2.05 | 6.89E-11 |
| Repeat_unit_range-77683..77713 | others | - | - | - | - | 2.83 | 1.22E-11 |
| Repeat_unit_range-84902..84961 | others | - | - | - | - | - | - |
| Repeat_unit_range-87442..87456 | others | - | - | - | - | - | - |
| Repeat_unit_range-87880..87918 | others | - | - | - | - | - | - |
| TR_repeat-unit_range69138..169671 | others | - | - | - | - | - | - |
| BHRF1_intron_acceptor_used_from_latent_transcripts | others | - | - | 0.89 | 1.46E-04 | 1.21 | 2.58E-07 |
| BLLF1-splice_variant | others | - | - | 1.04 | 5.66E-06 | 1.16 | 4.57E-07 |
| DRleft_similar_to42819..143875 | others | 0.97 | 1.96E-05 | 0.82 | 9.04E-05 | 1.14 | 1.02E-07 |
| DRright_similar_to_40265..41308 | others | 0.91 | 5.58E-05 | 0.87 | 3.36E-05 | 1.02 | 1.31E-06 |
| Dyad_Symmetry_EBNA1_Binding_site_II | others | - | - | - | - | - | - |
| EBNA_Binding_site_III | others | - | - | - | - | - | - |
| FR_Repeats_EBNA1_Binding_sites_I | others | - | - | - | - | - | - |

[a] Differentially enriched genes were identified by EdgeR analysis with FDR-q <0.05. "-": No significance;

[b] Gene types: different classes of EBV genes, including immediate-early (IE), early(E), late(L) and latent (LT) genes. Cp-promotor (Cp), Qp-promotor (Qp), ebv-miRNA(miR) and other regions are also shown.

**Supplementary Table 7. EBV genes methylation rates in coculture samples.**

| Genomic position [a] | | Annotated genes | Gene types[b] | Methylation rates (%) | | |
|---|---|---|---|---|---|---|
| Start | End | | | Untreated | *SSA* MOI=20 | *SSA* MOI=40 |
| 152866 | 153081 | BZLF1 | IE | 56.28 | 55.70 | 44.17 |
| 153167 | 153271 | BZLF1 | IE | 72.83 | 63.46 | 51.95 |
| 153423 | 153962 | BZLF1 | IE | 67.24 | 51.07 | 48.07 |
| 153423 | 155953 | BRLF1 | IE | 54.52 | 47.13 | 47.17 |
| 156894 | 156949 | BRLF1 | IE | 48.65 | 32.08 | 43.04 |
| 59 | 1653 | BGLF5 | E | 53.16 | 52.28 | 45.84 |
| 59 | 2926 | BGLF4 | E | 53.55 | 50.01 | 42.63 |
| 4791 | 8363 | BDLF4 | E | 47.35 | 40.80 | 39.55 |
| 16670 | 20283 | BcRF1 | E | 57.41 | 51.38 | 50.3 |
| 20205 | 24443 | BXLF1 | E | 60.79 | 53.11 | 49.35 |
| 32162 | 35304 | LF3 | E | 62.27 | 47.45 | 48.53 |
| 40616 | 42008 | LF2 | E | 57.34 | 48.43 | 42.39 |
| 40616 | 43285 | LF1 | E | 55.08 | 49.41 | 42.78 |
| 40616 | 44232 | BILF1 | E | 56.55 | 48.53 | 44.67 |
| 42963 | 47876 | BALF5 | E | 54.81 | 50.94 | 46.94 |
| 47832 | 52496 | BALF3 | E | 53.25 | 49.66 | 44.69 |
| 52138 | 55944 | BALF2 | E | 32.5 | 28.46 | 24.75 |
| 55976 | 56572 | BALF1 | E | 11.5 | 8.90 | 9.71 |
| 56596 | 57300 | BARF1 | E | 8.12 | 7.66 | 6.71 |
| 58070 | 58423 | BNLF2b | E | 4.57 | 2.69 | 3.42 |
| 58070 | 58654 | BNLF2a | E | 9.73 | 6.85 | 7.95 |
| 101283 | 103797 | BHLF1 | E | 57.86 | 44.70 | 46.97 |
| 104898 | 105315 | BHRF1 | E | 42.71 | 38.04 | 37.12 |
| 104739 | 106519 | BHRF1 | E | 46.88 | 42.01 | 40.33 |
| 106962 | 108064 | BFLF2 | E | 48.41 | 40.19 | 40.04 |
| 106962 | 109551 | BFLF1 | E | 42.78 | 37.62 | 36.12 |
| 110791 | 113057 | BFRF2 | E | 58.77 | 47.92 | 40.98 |
| 109815 | 113057 | BFRF1 | E | 54.08 | 47.92 | 40.98 |
| 127071 | 129790 | BORF2 | E | 48.41 | 45.21 | 41.69 |
| 130742 | 133106 | BMRF1 | E | 44.75 | 38.99 | 36.79 |
| 129706 | 133106 | BaRF1 | E | 49.16 | 38.99 | 36.79 |
| 133644 | 135023 | BSLF2/BMLF1 | E | 51.15 | 41.82 | 39.69 |
| 135130 | 135258 | BSLF2/BMLF1 | E | 57.98 | 51.64 | 42.25 |
| 133644 | 137783 | BSLF1 | E | 50.78 | 45.06 | 41.44 |
| 138510 | 139421 | BLLF3 | E | 42.57 | 39.60 | 35.63 |
| 140322 | 140953 | BLLF2 | E | 51.33 | 48.74 | 40.61 |
| 155784 | 156883 | BRRF1 | E | 39.42 | 44.66 | 46.14 |
| 160989 | 162428 | BKRF3 | E | 55.94 | 52.10 | 40.77 |

| | | | | | | |
|---|---|---|---|---|---|---|
| 162461 | 164895 | BBLF4 | E | 50.64 | 51.37 | 44.30 |
| 167328 | 168023 | BBLF2/BBLF3 | E | 50.9 | 48.25 | 36.80 |
| 168152 | 169675 | BBLF2/BBLF3 | E | 51.58 | 45.78 | 49.23 |
| 59 | 643 | BBLF1 | L | 50.8 | 52.21 | 49.02 |
| 59 | 3429 | BGLF3.5 | L | 54.24 | 50.09 | 42.23 |
| 59 | 4425 | BGLF3 | L | 53.92 | 49.8 | 42.76 |
| 4250 | 5185 | BGRF1/BDRF1 | L | 53.53 | 49.65 | 42.78 |
| 4791 | 6241 | BGLF2 | L | 54.38 | 45.73 | 45.97 |
| 4791 | 7741 | BGLF1 | L | 48.00 | 40.50 | 39.90 |
| 4791 | 8613 | BDLF3.5 | L | 47.95 | 41.08 | 40.52 |
| 8524 | 9661 | BGRF1/BDRF1 | L | 55.03 | 45.01 | 50.07 |
| 9663 | 10413 | BDLF3 | L | 59.43 | 46.84 | 48.26 |
| 9663 | 11785 | BDLF2 | L | 45.48 | 41.56 | 39.15 |
| 9663 | 12695 | BDLF1 | L | 44.61 | 41.32 | 39.45 |
| 12616 | 17019 | BcLF1 | L | 52.65 | 44.98 | 40.97 |
| 19013 | 20283 | BTRF1 | L | 62.88 | 51.38 | 50.30 |
| 20205 | 22618 | BXLF2 | L | 62.58 | 51.68 | 47.36 |
| 24168 | 26483 | BXRF1 | L | 53.56 | 50.08 | 47.79 |
| 24610 | 26483 | BVRF1 | L | 52.07 | 50.08 | 47.79 |
| 26229 | 27224 | BVLF1 | L | 54.33 | 56.4 | 48.66 |
| 27029 | 29052 | BVRF2 | L | 59.72 | 50.04 | 48.19 |
| 27928 | 29052 | BdRF1 | L | 60.52 | 50.04 | 48.19 |
| 29061 | 29879 | BILF2 | L | 61.34 | 51.56 | 43.48 |
| 47832 | 50500 | BALF4 | L | 55.61 | 53.44 | 48.12 |
| 65060 | 69228 | BNRF1 | L | 59.71 | 53.76 | 54.53 |
| 109507 | 113057 | BFRF1A | L | 54.12 | 47.92 | 40.98 |
| 112490 | 113057 | BFRF3 | L | 44.35 | 47.92 | 40.98 |
| 113046 | 123091 | BPLF1 | L | 46.28 | 41.56 | 39.08 |
| 113046 | 126224 | BOLF1 | L | 49.12 | 42.34 | 39.61 |
| 125919 | 129790 | BORF1 | L | 48.10 | 45.21 | 41.69 |
| 131681 | 133106 | BMRF2 | L | 49.95 | 38.99 | 36.79 |
| 137784 | 138506 | BSRF1 | L | 48.93 | 44.78 | 39.50 |
| 139409 | 140319 | BLRF1 | L | 45.73 | 45.42 | 40.79 |
| 139765 | 140319 | BLRF2 | L | 47.8 | 45.42 | 40.79 |
| 140322 | 142941 | BLLF1 | L | 44.94 | 37.91 | 38.42 |
| 152185 | 152856 | BZLF2 | L | 57.28 | 47.95 | 41.21 |
| 157011 | 158687 | BRRF2 | L | 44.99 | 42.99 | 41.89 |
| 161770 | 162428 | BKRF4 | L | 57.40 | 52.10 | 40.77 |
| 160541 | 162428 | BKRF2 | L | 55.43 | 52.10 | 40.77 |
| 166584 | 167426 | BBRF2 | L | 45.41 | 45.24 | 35.53 |
| 164512 | 167426 | BBRF1 | L | 49.24 | 45.24 | 35.53 |

| | | | | | | |
|---|---|---|---|---|---|---|
| 169617 | 170999 | BBRF3 | L | 53.08 | 51.28 | 46.47 |
| 29949 | 30077 | RPMS1 | LT | 54.43 | 40.66 | 42.59 |
| 31039 | 31144 | RPMS1 | LT | 62.04 | 51.29 | 43.83 |
| 32150 | 32283 | RPMS1 | LT | 84.31 | 53.44 | 54.39 |
| 41172 | 41303 | RPMS1 | LT | 46.07 | 48.52 | 41.67 |
| 41514 | 41668 | RPMS1 | LT | 64.34 | 47.48 | 43.51 |
| 41828 | 41939 | RPMS1 | LT | 56.74 | 46.76 | 37.71 |
| 46855 | 48325 | RPMS1 | LT | 52.46 | 48.21 | 48.80 |
| 47137 | 48325 | A73 | LT | 50.62 | 48.21 | 48.80 |
| 48434 | 48516 | A73 | LT | 49.09 | 53.08 | 52.66 |
| 50213 | 50339 | A73 | LT | 62.64 | 58.81 | 49.71 |
| 51369 | 52119 | A73 | LT | 39.68 | 27.28 | 22.97 |
| 50213 | 52119 | RPMS1 | LT | 51.28 | 27.28 | 22.97 |
| 50709 | 52124 | BARF0 | LT | 47.48 | 35.02 | 30.25 |
| 57627 | 58045 | LMP-2A | LT | 3.17 | 3.57 | 4.17 |
| 58070 | 60049 | LMP-1 | LT | 16.72 | 13.98 | 13.57 |
| 60127 | 60213 | LMP-1 | LT | 12.16 | 15.70 | 14.67 |
| 60292 | 60599 | LMP-1 | LT | 3.37 | 4.52 | 4.35 |
| 60837 | 60991 | LMP-2B | LT | 9.12 | 9.01 | 11.13 |
| 63427 | 63641 | LMP-2B | LT | 80.35 | 78.61 | 65.42 |
| 63729 | 63827 | LMP-2B | LT | 76.32 | 68.55 | 54.71 |
| 63909 | 64157 | LMP-2B | LT | 68.88 | 64.25 | 55.72 |
| 64240 | 64320 | LMP-2B | LT | 68.54 | 58.39 | 60.08 |
| 64395 | 64565 | LMP-2B | LT | 78.99 | 65.48 | 65.06 |
| 64649 | 64864 | LMP-2B | LT | 72.26 | 63.57 | 59.92 |
| 64943 | 65051 | LMP-2B | LT | 55.66 | 51.86 | 56.56 |
| 68777 | 69228 | LMP-2B | LT | 71.59 | 53.76 | 54.53 |
| 98746 | 98778 | EBNA-LP | LT | 7.01 | 6.21 | 5.11 |
| 98863 | 98984 | EBNA-LP | LT | 47.78 | 48.26 | 41.43 |
| 99371 | 99429 | EBNA-LP | LT | 12.15 | 3.79 | 2.86 |
| 100924 | 101014 | EBNA-LP | LT | 22.45 | 16.67 | 39.57 |
| 74601 | 74744 | Cp-EBNA2 | Cp | 53.7 | 47.66 | 53.81 |
| 74891 | 74922 | Cp-EBNA2 | Cp | 68.24 | 59.48 | 49.30 |
| 99371 | 101014 | Cp-EBNA2 | Cp | 57.23 | 16.67 | 39.57 |
| 142987 | 143330 | Cp-EBNA3A | Cp | 57.49 | 43.3 | 48.85 |
| 143419 | 145999 | Cp-EBNA3A | Cp | 45.52 | 45.58 | 42.10 |
| 146057 | 146458 | Cp-EBNA3B | Cp | 58.27 | 50.43 | 44.02 |
| 149053 | 149419 | Cp-EBNA3C | Cp | 55.52 | 44.75 | 42.08 |
| 146537 | 152510 | Cp-EBNA3B | Cp | 51.87 | 46.24 | 43.58 |
| 149494 | 152510 | Cp-EBNA3C | Cp | 55.37 | 46.24 | 43.58 |
| 158710 | 160578 | Qp-EBNA1 | Cp | 49.99 | 42.77 | 40.77 |

| | | | | | | |
|---|---|---|---|---|---|---|
| 113405 | 113441 | Qp-EBNA1 | Qp | 2.45 | 1.41 | 2.50 |
| 118461 | 118632 | Qp-EBNA1 | Qp | 55.51 | 37.81 | 41.91 |
| 30858 | 30880 | ebv-miR-BART4* | miR | 88.89 | 59.68 | 57.14 |
| 30943 | 30966 | ebv-miR-BART1-5p | miR | 65.91 | 53.76 | 53.57 |
| 30979 | 31000 | ebv-miR-BART1-3p | miR | 73.47 | 51.35 | 48.82 |
| 31268 | 31291 | ebv-miR-BART5 | miR | 56.92 | 52.48 | 42.11 |
| 31310 | 31327 | ebv-miR-BART5* | miR | 55.74 | 52.74 | 43.48 |
| 31508 | 31529 | ebv-miR-BART17-5p | miR | 48.84 | 57.29 | 43.68 |
| 37591 | 37612 | ebv-miR-BART18-3p | miR | 66.04 | 55.28 | 43.36 |
| 38361 | 38382 | ebv-miR-BART8 | miR | 58.18 | 44.76 | 50.00 |
| 38396 | 38418 | ebv-miR-BART8* | miR | 40.43 | 33.82 | 42.20 |
| 38586 | 38608 | ebv-miR-BART9 | miR | 46.81 | 38.61 | 41.96 |
| 39127 | 39150 | ebv-miR-BART11-5p | miR | 58.14 | 47.00 | 58.88 |
| 39165 | 39185 | ebv-miR-BART11-3p | miR | 69.77 | 53.85 | 52.53 |
| 39806 | 39828 | ebv-miR-BART19-5p | miR | 58.54 | 53.01 | 35.29 |
| 40117 | 40138 | ebv-miR-BART13* | miR | 66.93 | 44.07 | 48.15 |
| 40154 | 40176 | ebv-miR-BART13 | miR | 75.61 | 55.66 | 45.31 |
| 40335 | 40356 | ebv-miR-BART14* | miR | 66.67 | 60.48 | 50.31 |
| 44338 | 44359 | ebv-miR-BART2-5p | miR | 43.48 | 44.00 | 55.68 |
| 44374 | 44397 | ebv-miR-BART2-3p | miR | 47.37 | 45.83 | 53.85 |
| 104742 | 104763 | ebv-miR-BHRF1-1 | miR | 50.00 | 38.61 | 41.04 |
| 106156 | 106177 | ebv-miR-BHRF1-2 | miR | 61.22 | 54.55 | 56.20 |
| 106236 | 106257 | ebv-miR-BHRF1-3 | miR | 60.42 | 38.30 | 45.86 |
| 30544 | 30921 | Repeat_region | others | 75.21 | 52.40 | 52.92 |
| 31664 | 31685 | ebv-miR-BART6-3p | others | 64.44 | 54.72 | 50.00 |
| 32357 | 34874 | Repeat_IR4_PstI | others | 70.44 | 43.17 | 52.26 |
| 34865 | 35921 | DRright_similar_to_40265..41308 | others | 51.04 | 45.88 | 41.61 |
| 34800 | 36037 | oriLyt | others | 52.13 | 47.00 | 43.40 |
| 59497 | 59658 | Repeat_unit_range_167452..167484 | others | 2.90 | 3.85 | 0.99 |
| 61184 | 63319 | TR_repeat-unit_range_169138..169671 | others | 32.40 | 23.33 | 26.05 |
| 70005 | 70171 | EBER1 | others | 1.09 | 3.54 | 4.41 |
| 70332 | 70505 | EBER2 | others | 1.58 | 5.39 | 3.41 |
| 70798 | 71307 | FR_Repeats_EBNA1_Binding_sites_I | others | 4.04 | 1.30 | 0.41 |
| 72286 | 72400 | Dyad_Symmetry_EBNA1_Binding_site_II | others | 2.36 | 0.41 | 0.85 |
| 70692 | 72577 | OriP | others | 12.51 | 11.56 | 12.61 |

| | | | | | | |
|---|---|---|---|---|---|---|
| 72896 | 73527 | BCRF1/IL10 | others | 56.74 | 48.55 | 46.42 |
| 75266 | 98628 | IR1_W_repeats | others | 15.05 | 22.25 | 21.94 |
| 99663 | 99782 | Repeat_unit_range-36294..36302 | others | 10.00 | 0 | 12.50 |
| 101559 | 103096 | IR2/NotI_repeats | others | 40.22 | 51.48 | 60.11 |
| 103569 | 104561 | OriLyt | others | 62.53 | 42.21 | 43.64 |
| 103634 | 104677 | DRleft_similar_to_142819..143875 | others | 57.66 | 39.11 | 38.97 |
| 113413 | 113460 | EBNA_1_Binding_site_III | others | 2.27 | 1.68 | 2.85 |
| 120667 | 120913 | Repeat_unit_range-57298..57348 | others | 51.56 | 41.32 | 39.91 |
| 121370 | 121414 | Repeat_unit_range-58001..58015 | others | 59.46 | 51.15 | 36.36 |
| 133221 | 133363 | Repeat_unit_range-69852..69922 | others | 24.18 | 25.27 | 25.40 |
| 134542 | 134631 | Repeat_unit_range-71173..71181 | others | 21.78 | 22.53 | 23.00 |
| 140322 | 140963 | BLLF1-splice_variant | others | 51.33 | 48.74 | 40.61 |
| 141402 | 142941 | BLLF1-splice_variant | others | 41.35 | 37.91 | 38.42 |
| 144957 | 145026 | Repeat_family_type_A | others | 46.15 | 20.37 | 16.28 |
| 145030 | 145055 | Repeat_family_type_B | others | 60.00 | 50.94 | 52.83 |
| 145056 | 145130 | Repeat_family_type_C | others | 46.39 | 41.09 | 52.92 |
| 145135 | 145160 | Repeat_family_type_B | others | 41.82 | 46.9 | 54.24 |
| 145161 | 145238 | Repeat_family_type_C | others | 48.89 | 53.46 | 50.00 |
| 145239 | 145309 | Repeat_family_type_A | others | 47.06 | 50.30 | 44.40 |
| 145320 | 145397 | Repeat_family_type_C | others | 49.12 | 54.48 | 45.00 |
| 145398 | 145468 | Repeat_family_type_A | others | 41.56 | 45.79 | 45.96 |
| 145645 | 145731 | Repeat_family_type_D | others | 21.15 | 8.65 | 3.64 |
| 145732 | 145818 | Repeat_family_type_D | others | 4.84 | 11.54 | 6.70 |
| 148271 | 148387 | Repeat_unit_range-84902..84961 | others | 49.58 | 59.70 | 45.08 |
| 150811 | 150993 | Repeat_unit_range-87442..87456 | others | 59.49 | 52.72 | 50.60 |
| 151249 | 151521 | Repeat_unit_range-87880..87918 | others | 53.23 | 50.19 | 41.83 |
| 153322 | 153420 | Repeat_region | others | 86.52 | 54.48 | 51.42 |
| 158985 | 159620 | Repeat_family-IR3 | others | 49.11 | 43.99 | 38.25 |

[a] CpG start and end position annotated in EBV genome;

[b] Gene types: different classes of EBV genes, including immediate-early (IE), early(E), late(L) and latent (LT) genes. Cp-promotor (Cp), Qp-promotor (Qp), ebv-miRNA(miR) and other regions are also shown.

**Supplementary Table 8. Primers for identifying the species of *Streptococcus*_ASV.1b51.**

| Species | Primers | Primer sequences (5'-3') | Gene | References |
|---|---|---|---|---|
| *Streptococcus sanguinis* | S.sanguinis-F | GGTTAATGCCGATAATGCGATG | / | Lee *et al.*[1] |
| | S.sanguinis-R | CGGCTCATATCGTAAATTCCAATG | | |
| *Streptococcus cristatus* | S.cristatus-F | CTGACGAAGCGAAAGGTCTG | *arc*A | Wang *et al.*[2] |
| | S.cristatus-R | ATGTGGTTGAGCGATACAGC | | |
| *Streptococcus sinensis* | S.sinensis-F | TAGTTTACTACACCGTAC | *16S rRNA* | Wong *et al.*[3] |
| | S.sinensis-R | CTTACCATGCAGTAAGAT | | |
| *Streptococcus gordonii* | S.gordonii-F | CCTTGGAGCAAGGAATATTTTGAATCTG | *gdh* | Banas *et al.*[4] |
| | S.gordonii-R | CTTCTTGGCTCGGTTGTGTCAAGCG | | |
| *Streptococcus parasanguinis* | Spa146f | AACAATGCGATYCCAGTATCRAG | *GroEL* | Chen *et al.*[5] |
| | Spa525r | CTACGACATTAAAGGTACCDCGG | | |

**Supplementary Table 9. EBV specific primers and probes used in this study.**

| Genes | Primers | Primer sequences (5'-3') | References |
|---|---|---|---|
| **EBV DNA load** | | | |
| BamHI-W | BamHI-W-F | CCCAACACTCCACCACACC | WQ Xue *et al.*[6] |
| | BamHI-W-R | TCTTAGGAGCTGTCCGAGGG | |
| | BamHI-W-probe | CACACACTACACACACCCACCCGTCTC | |
| β-globin | β-globin-F | GTGCACCTGACTCCTGAGGAGA | WQ Xue *et al.*[6] |
| | β-globin-R | CCTTGATACCAACCTGCCCAG | |
| | β-globin-probe | AAGGTGAACGTGGATGAAGTTGGTGG | |
| **EBV mRNA profile** | | | |
| *BRLF1* | BRLF1-F | CGAGGACGGGATAGGTGAAC | L Hu *et al.*[7] |
| | BRLF1-R | CGGCAAGCAGGTAGTGGAAC | |
| *BMRF1* | BMRF1-F | TCTCAAGGGAGGAGTGCTGC | L Hu *et al.*[7] |
| | BMRF1-R | TCTGGGCTCTGGTGATTCTG | |
| *BLLF1* | BLLF1-F | ACACTCATTATCACACGAACGG | L Hu *et al.*[7] |
| | BLLF1-R | AGTAGAGCTGGGTAGACCTGTC | |
| *BZLF2* | BZLF2-F | TGATGTCCAGTTCCCTTCTCC | L Hu *et al.*[7] |
| | BZLF2-R | GGTGTTTCCAAGCCTGTGC | |
| *BXLF2* | BXLF2-F | GATGGGTTTCTTGGGCGTCTC | L Hu *et al.*[7] |
| | BXLF2-R | GCCACAGCACCCTGCGAAC | |
| *BKRF2* | BKRF2-F | GCCACCTGTCTTGTCACCATT | L Hu *et al.*[7] |
| | BKRF2-R | TTGCTCACCAGGTAAATGTCG | |
| *LMP1* | LMP1-F | GTATTGGCACAAGATGGAAAGC | L Hu *et al.*[7] |
| | LMP1-R | CAACTACCAGGCAGATGAGGC | |
| *LMP2A* | LMP2A-F | ACGATGGCGGAAACAACTC | L Hu *et al.*[7] |
| | LMP2A-R | GGGTCCTCATAAGGCGGTG | |
| *EBNA1* | EBNA1-F | GTAGGGGATGCCGATTATTTTG | L Hu *et al.*[7] |
| | EBNA1-R | CTCCTTGACCACGATGCTTTC | |
| *BALF4* | BALF4-F | AACCTTTGACTCGACCATCG | Atsuko Sugimoto |
| | BALF4-R | ACCTGCTCTTCGATGCACTT | *et al.*[8] |
| **Houskeeping gene** | | | |
| GAPDH | GAPDH-F | CTCCTCCTGTTCGACAGTCAGC | |
| | GAPDH-R | CCCAATACGACCAAATCCGTT | |

## References


1. Lee, J.W., J.Y. Jung, and S.K. Lim, *Simple and rapid identification of saliva by detection of oral streptococci using direct polymerase chain reaction combined with an immunochromatographic strip.* Forensic Sci Int Genet, 2018. **33**: p. 155-160.
2. Wang, B.Y., et al., *Negative correlation of distributions of Streptococcus cristatus and Porphyromonas gingivalis in subgingival plaque.* J Clin Microbiol, 2009. **47**(12): p. 3902-6.
3. Woo, P.C., et al., *The oral cavity as a natural reservoir for Streptococcus sinensis.* Clin Microbiol Infect, 2008. **14**(11): p. 1075-9.
4. Banas, J.A., et al., *PCR-Based Identification of Oral Streptococcal Species.* Int J Dent, 2016. **2016**: p. 3465163.
5. Chen, Q., et al., *Quantification of Human Oral and Fecal Streptococcus parasanguinis by Use of Quantitative Real-Time PCR Targeting the groEL Gene.* Front Microbiol, 2019. **10**: p. 2910.
6. Xue, W.Q., et al., *Decreased oral Epstein-Barr virus DNA loads in patients with nasopharyngeal carcinoma in Southern China: A case-control and a family-based study.* Cancer Med, 2018. **7**(7): p. 3453-64.
7. Hu, L., et al., *Comprehensive profiling of EBV gene expression in nasopharyngeal carcinoma through paired-end transcriptome sequencing.* Front Med, 2016. **10**(1): p. 61-75.
8. Sugimoto, A., et al., *Different distributions of Epstein-Barr virus early and late gene transcripts within viral replication compartments.* J Virol, 2013. **87**(12): p. 6693-9.
